# Age of heart disease presentation and dysmorphic nuclei in patients with LMNA mutations

**DOI:** 10.1371/journal.pone.0188256

**Published:** 2017-11-17

**Authors:** Jason Q. Core, Mehrsa Mehrabi, Zachery R. Robinson, Alexander R. Ochs, Linda A. McCarthy, Michael V. Zaragoza, Anna Grosberg

**Affiliations:** 1 Departments of Biomedical Engineering, University of California, Irvine, CA, United States of America; 2 The Edwards Lifesciences Center for Advanced Cardiovascular Technology, University of California, Irvine, CA, United States of America; 3 Pediatrics–Genetics & Genomics Division–School of Medicine, University of California, Irvine, CA, United States of America; 4 Biological Chemistry–School of Medicine, University of California, Irvine, CA, United States of America; 5 Chemical Engineering and Materials Science, University of California, Irvine, CA, United States of America; Rutgers University Newark, UNITED STATES

## Abstract

Nuclear shape defects are a distinguishing characteristic in laminopathies, cancers, and other pathologies. Correlating these defects to the symptoms, mechanisms, and progression of disease requires unbiased, quantitative, and high-throughput means of quantifying nuclear morphology. To accomplish this, we developed a method of automatically segmenting fluorescently stained nuclei in 2D microscopy images and then classifying them as normal or dysmorphic based on three geometric features of the nucleus using a package of Matlab codes. As a test case, cultured skin-fibroblast nuclei of individuals possessing *LMNA* splice-site mutation (c.357-2A>G), *LMNA* nonsense mutation (c.736 C>T, pQ246X) in exon 4, *LMNA* missense mutation (c.1003C>T, pR335W) in exon 6, Hutchinson-Gilford Progeria Syndrome, and no LMNA mutations were analyzed. For each cell type, the percentage of dysmorphic nuclei, and other morphological features such as average nuclear area and average eccentricity were obtained. Compared to blind observers, our procedure implemented in Matlab codes possessed similar accuracy to manual counting of dysmorphic nuclei while being significantly more consistent. The automatic quantification of nuclear defects revealed a correlation between in vitro results and age of patients for initial symptom onset. Our results demonstrate the method’s utility in experimental studies of diseases affecting nuclear shape through automated, unbiased, and accurate identification of dysmorphic nuclei.

## Introduction

Abnormal cellular structures arising from proteins expressed by mutant genes can negatively impact tissue and organ function predisposing individuals inheriting genetic mutations to disease [1–3]. One particularly severe example is Hutchinson-Gilford Progeria Syndrome (HGPS) in which a mutation in the Lamin A/C (*LMNA*) gene causes premature aging up to seven times faster than normal, usually culminating in death due to cardiovascular complications [1]. Other types of *LMNA* gene mutations are associated with a range of heart disease symptoms including cardiomyopathy, heart failure, and arrhythmia [2,4,5]. While the exact mechanism by which *LMNA* mutations cause heart disease is under investigation, it is known that the *LMNA* gene expresses both the Lamin A and C proteins via alternative splicing. These proteins form the integral meshes of the nuclear lamina, which provides structural support to the nucleus [6]. Consequently, deficiencies in the nuclear lamina have been shown to perturb nuclear shape by, for example, causing the formation of blebs [7]. Indeed, in some patients, different cell types will exhibit varied nuclear envelope imperfections such as large defects in cardiomyocyte nuclei and the fibroblast defects [8]. This is especially interesting as some nuclear envelope protein mutations lead to organ specific pathologies in patients. For example, there are a variety of *LMNA* mutations including heterozygous splice site, nonsense, and missense mutation, which cause diverse heart diseases with no other major pathologies [4,9,10]. Furthermore, even patients with the same mutation can exhibit a variety of heart disease symptoms with different presentation ages from 36–54 years [4]. So far, it has not been shown if subtle differences exist in fibroblasts of these patients that would be informative of the variability among the patients with the same mutation. *LMNA* mutations are widely hypothesized to alter the mechanical properties of cell nuclei through the production of defective structural proteins, thus compromising the functionality and health of certain cell types [2,11–13]. Alterations in nuclear shape have been correlated with changes in cellular processes such as gene expression and cell viability [12–15]. Nuclear deformity is therefore a pertinent deficiency in cell structure to be considered in investigations patient variability.

While dysmorphic nuclei are a hallmark of many diseases such as laminopathies [7,13] and certain types of cancer [16–18], both unaffected and diseased cell populations can exhibit nuclear shape defects [19,20]. Indeed, nuclei of some cell types are not universally spheroid or elliptical, and frequently possess folds, protrusions, and other disturbances in boundary smoothness likely involved in normal biological processes [21–24]. This makes the detection of additional nuclear defects caused by disease far more difficult. Moreover, the proportion of nuclei identified as abnormally shaped through manual observation of either diseased or unaffected cells is subjective and user-dependent. Automatic, unbiased detection of dysmorphic nuclei by image processing software is, therefore, preferable for both effectively quantifying nuclear shape abnormality and determining the extent to which it arises from pathology.

Many existing methods of automatically detecting irregularities in nuclear morphology calculate a single feature related to nuclear shape, measuring nuclear defect levels as the difference in mean values of the feature between entire cell populations. Examples include quantifying nuclear shape asymmetry averages [25] or the degree of negative boundary curvature averaged over entire cell populations [20]. However, such approaches which attempt to capture nuclear imperfections as an average of a single morphometric feature across a large number of cells cannot identify individual nuclei as normally or abnormally shaped, or determine the proportion of dysmorphic nuclei in a tissue. Machine learning is sometimes utilized for the classification of disease states based on nuclear morphology, requiring a multitude (>100) of shape features [25]. As with any machine learning algorithm, this is challenging to replicate without performing re-training and re-validation on each experimental setup. A similar multi-parametric, automated approach to describing nuclear morphology using far fewer features could be used to designate single nuclei as normal or dysmorphic and evaluate the percentage of defective nuclei within an entire population, a simpler, more intuitive, and more easily implemented measure of nuclear defect levels.

In this work, we have developed a method of automatically segmenting nuclei in two-dimensional fluorescent images and classifying each as possessing a normal or dysmorphic morphology based on three nuclear shape features by a package of Matlab codes. As proof of concept, this technique was used to quantify the prevalence of dysmorphic nuclei in skin fibroblasts of individuals with three different *LMNA* mutations (LMNA-CMs), unaffected family members and unrelated individuals as negative controls (Controls), and a patient with Hutchinson-Gilford Progeria Syndrome as a positive control (HGPS). The developed method identifies dysmorphic nuclei with accuracy comparable to manual observation in a significantly more consistent and unbiased manner and allowed for correlating of subtle differences in nuclear shape of fibroblasts to a phenotype the patients presented in the clinic.

## Materials & methods

### Substrate fabrication

A 7.6cm x 8.3cm rectangular glass coverslip (Fisher Scientific Company, Hanover Park, IL) was cleaned via sonication for 30 minutes in 200 proof ethanol solution. The coverslip was then spin-coated with a 10:1 mixture of polydimethylsiloxane (PDMS) and curing agent (PDMS, Ellsworth Adhesives, Germantown, WI), and cured at 65°C for at least 12 hours. Finally, the large coverslip was cut into smaller, rectangular 14mm x 12.5mm coverslips using a diamond scriber (VWR, Radnor, PA). Coverslips were placed PDMS side facing downward onto 100 μL drops of 0.05 mg/mL fibronectin solution for 10 minutes. Coverslips were then rinsed 3 times in Phosphate Buffered Saline (PBS, Life Technologies, Carlsbad, CA) and stored in 4°C PBS until seeded with cells.

### Skin fibroblast collection

Human fibroblast cells were collected from three families with different mutations of heterozygous *LMNA* splice-site mutation (c.357-2A>G) [4] (Family A); *LMNA* nonsense mutation (c.736 C>T, pQ246X) in exon 4 [10] (Family B); *LMNA* missense mutation (c.1003C>T, pR335W) in exon 6 [9] (Family C). Moreover, related individuals’ fibroblast cells in each family were collected as mutation-negative controls. Unrelated negative control fibroblast cells were purchased from Lonza (catalog# CC-2511) and Coriell (catalog# ND31845, AG14284). Informed consent for these studies was performed in accordance with the UC Irvine Institutional Review Board, which specially approved this study (IRB# 2014–1253). For positive control, HGPS fibroblast cells were obtained from the Coriell Institute for Medical Research (Camden, New Jersey: catalog #AG11513). In this case, the fibroblasts were grown from a skin biopsy taken from an 8-year-old female HGPS patient possessing a *LMNA* G608G point mutation [26]. Table 1 summarizes the cell sources and the abbreviations used for each group in this manuscript.

**Table 1:** This table contains the different experimental groups (cell sources), the abbreviations used to describe each group in the manuscript, and the passage number of the cells from that group.

Cell Sources	Abbreviation	Passage #
Patients in families with *LMNA* mutations, but only heart disease as the main pathology	LMNA-CM	7
The family in which some of the member have splice-site *LMNA* mutation	Family A	7
Individuals with the mutation from family A (Patients from family A)	LMNA-CM-A	7
The family in which some of the member have Nonsense *LMNA* mutation	Family B	7
Individuals with the mutation from family B (Patients from family B)	LMNA-CM-B	7
The family in which some of the member have Missense *LMNA* mutation	Family C	7
Individuals with the mutation from family C (Patients from family C)	LMNA-CM-C	7
Hutchinson-Gilford *progeria* syndrome (purchased from Coriell Institute for Medical Research)	HGPS	16
Siblings of patients from families A-C with no *LMNA* mutations (Related Negative Control)	R.N.-CONTROL	7
Unrelated Negative Control (commercially purchased cell lines)	U.N.-CONTROL	7
Aggregated Negative Control (Both Related and Unrelated Negative controls aggregated into a single group)	Neg-CONTROL	7

### Cell culture

LMNA-CM patient and Neg-CONTROL lines were expanded to passage 7 for experiments. HGPS cell lines were expanded to passage 16. At 80% to 100% confluency, the cells were passaged using 0.05% Trypsin.

Cells were seeded on 4–12 isotropic coverslips at the desired passage number and optimal density. Cells were then expanded in Minimum Essential Media (MEM) with 10% Fetal Bovine Serum (FBS, Life Technologies, Carlsbad, CA) and 1% Antibiotic-Antimycotic (Thermofisher Scientific, Waltham, MA). At 24 hours of incubation, the media was changed to MEM with 2% FBS. After an additional 24 hours of incubation, the cells were fixed.

As the division rate depended on patient age, the optimal cell seeding density was determined by seeding cells on isotropic coverslips in amounts varying from 75,000 to 400,000 cells per coverslip, and expanding them as described above. After 48 hours of incubation, these coverslips were examined and the optimal cell seeding density for confluency at 2 days was identified.

### Fixing and immunofluorescent staining

Staining was performed using standard techniques [27]. Briefly, upon completion of the cell culture period, media was aspirated and coverslips were rinsed 3 times in 2–3 mL of warmed PBS. Cells were then incubated in a warm 4% paraformaldehyde (PFA) solution (Fisher Scientific Company, Hanover Park, IL) containing 0.0005% Triton X-100 (Sigma Aldrich Inc., Saint Louis, MO) for 10 minutes at room temperature. Following fixation, the coverslips were again washed 3 times in 2–3 mL of warmed PBS, allowing 5 minutes between rinses. The fixed samples were then stained for nuclei (4’,6’-diaminodino-2-phenylinodole (DAPI), Life Technologies, Carlsbad, CA), actin (Alexa Fluor 488 Phalloidin, Life Technologies, Carlsbad, CA), and either fibronectin (polycloncal rabbit anti-human fibronectin, Sigma Aldrich Inc., Saint Louis, MO) or Lamin A/C (rabbit monoclonal EP4520 (ab133256) Anti-Lamin A+C, Abcam, San Fransisco, CA). Coverslips were then rinsed 3 times in PBS to prevent background staining. Secondary staining of either fibronectin or Lamin A/C was achieved using goat anti-rabbit IgG antibodies (Alexa Fluor 750 goat anti-rabbit, Life Technologies, Carlsbad, CA). The coverslips were washed in PBS to remove residual stain and mounted on glass microscope slides with room temperature Prolong Gold Antifade Mountant (Life Technologies, Carlsbad, CA) to prevent fading of fluorescent stains during microscopy. Finally, a commercial, clear nail polish was applied as a sealant along the edges of coverslips and then allowed to dry for 24 hours.

### Image acquisition

The microscope slides containing the stained samples were imaged using an UPLFLN 40x oil immersion objective (Olympus America, Center Valley, PA) and digital CCD camera ORCA-R2 C10600-10B (Hamamatsu Photonics, Shizuoka Prefecture, Japan) mounted on an IX-83 inverted motorized microscope (Olympus America, Center Valley, PA). Fluorescence images of ten randomly selected fields of view per slide were taken at 40x magnification (6.22 pixels/μm resolution) for each sample.

### Image analysis and feature extraction from nuclear boundary

**Fig 1:**
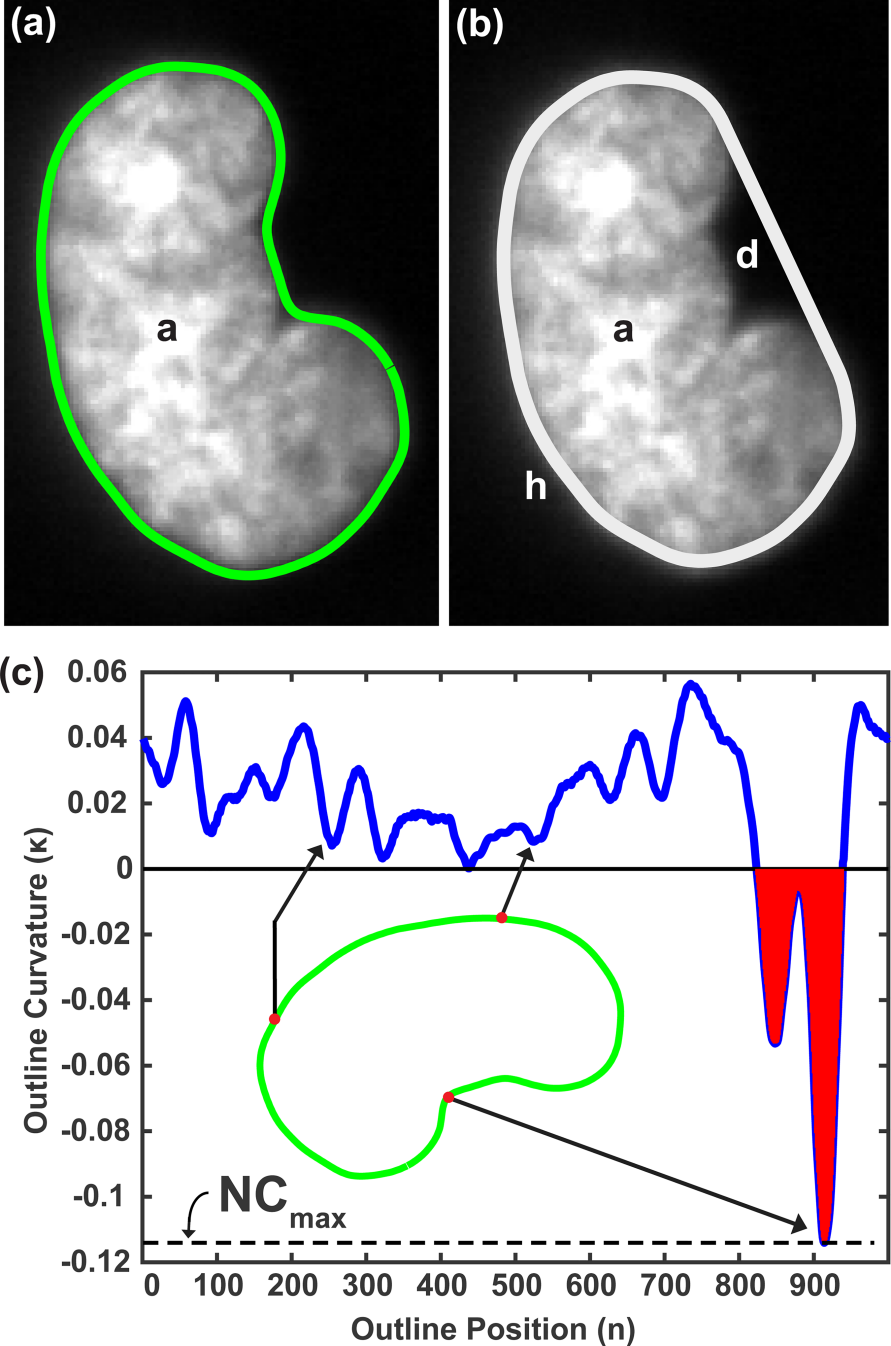
Quantifying nuclear shape characteristics. A) Nucleus outline generated by MATLAB image analysis, where *a* = nuclear cross sectional area; B) Convex hull generated from nucleus outline, where *h* = area of convex hull, *d = h–a*; C) Curvature profile of nucleus boundary. Arrows beginning at points along the nucleus boundary show the curvature value corresponding to that boundary point. Mean negative curvature is calculated using the average of all negative curvature values (shaded in red) (Eq 2). The point of maximum negative curvature is indicated by a dotted line.

Fluorescently labeled nuclei were detected and outlined using custom-written Matlab codes. A detailed description of the image analysis process is provided in the Supplemental Materials (S1 Fig and S1 Text). Briefly, each nucleus was isolated (S4C and S4D Fig), individually thresholded (S4E Fig), and outlined with the snake algorithm (S4F and S4G Fig). The natural coordinates of the outline were then interpolated to consist of 1000 evenly-spaced points (S4G Fig). In order to classify nuclei as normal or dysmorphic, three shape features related to the nuclear boundary were calculated. First, relative concavity (RC) of each nucleus (Fig 1A) was calculated using the method described in Langevin, et al. [28]. The difference (*d*) between the area of the convex hull (*h*) of each nucleus and the area enclosed by its calculated boundary (*a*) was divided by the convex hull area (Fig 1B):

$$RC=\frac{h-a}{h}=\frac{d}{h}$$ (1)

Next, mean negative curvature (MNC) was calculated for each nucleus boundary using the method described in Driscoll, et al. [20]. The extrinsic curvature of each point along the nucleus boundary was calculated by fitting a circle (S4 Code) to that boundary point and two other points each 25 boundary points to each side of it, and then using the radius to calculate curvature as *κ = $\frac{1}{r}$*. All negative curvature values along the boundary of a nucleus were averaged, and then multiplied by the square root of the area of that nucleus in order to non-dimensionalize the parameter:

$$MNC=\sqrt{a}\frac{\sum_{l=1}^{l=N}\left[(\frac{\kappa_{l}}{\left|\kappa_{l}\right|}-1)*\frac{1}{2}*\kappa_{l}\right]}{\sum_{l=1}^{l=N}\left[(1-\frac{\kappa_{l}}{\left|\kappa_{l}\right|})*\frac{1}{2}\right]}=-\sqrt{a}\frac{\sum_{l=1}^{l=N}\left[\frac{\kappa_{l}^{2}-\kappa_{l}|\kappa_{l}|}{\left|\kappa_{l}\right|}\right]}{\sum_{l=1}^{l=N}\left[\frac{\kappa_{l}-|\kappa_{l}|}{\left|\kappa_{l}\right|}\right]}$$ (2)

where *l* represents a nucleus boundary position, and N is the total number of boundary points. Calculation of mean negative curvature according to Eq 2 was accomplished in Matlab logically (using the *find* function to group all boundary curvature values below zero) rather than algebraically. Areas of negative curvature correspond to both blebs and invaginations in the nucleus due to the inward folding of the nuclear envelope, creating regions where the nuclear boundary becomes concave as opposed to the positive boundary curvature created by the smooth surface of a normal nucleus (Fig 1C). Any nucleus possessing no points of negative boundary curvature was assigned a mean negative curvature value of zero. Lastly, the single most negative curvature value (*NC*_*max*_) in the boundary curvature profile of each nucleus was also recorded and used as a feature to assess nuclear shape abnormality (Fig 1C):

$${NC}_{max}=\underset{1\leq l\leq N}{\max}\;\sqrt{a}(\frac{1}{2}\left(\frac{\kappa_{l}^{2}-\kappa_{l}|\kappa_{l}|}{\left|\kappa_{l}\right|}\right))$$ (3)

To better elucidate observer-code and observer-observer differences, three dimensional (3D) plots were generated with the RC, MNC, and NC_max_ variables on the axes. On the 3D plot, each nucleus in the validation set corresponded to a single point color coded according to agreements (agreement on normal, disagreement (normal/defective), disagreement (defective/normal), and agreement on defective).

### Statistics

Analysis of Variance (ANOVA) with Tukey’s method was used for mean comparisons among conditions. The student t-test was used if comparison was made between a single pair of data points. Linear trends were determined using linear regression, and both the R^2^ value for the trend and significance for both constants were calculated. P values of 0.05 or less were considered statistically significant. The level of agreement in classification of nuclei among different manual observers and the Matlab analysis codes was assessed using percent agreement, but Cohen’s Kappa was also calculated as it was possible for the manual observers to guess [29,30].

## 3. Results

### Automatic designation of abnormally shaped nuclei

**Fig 2:**
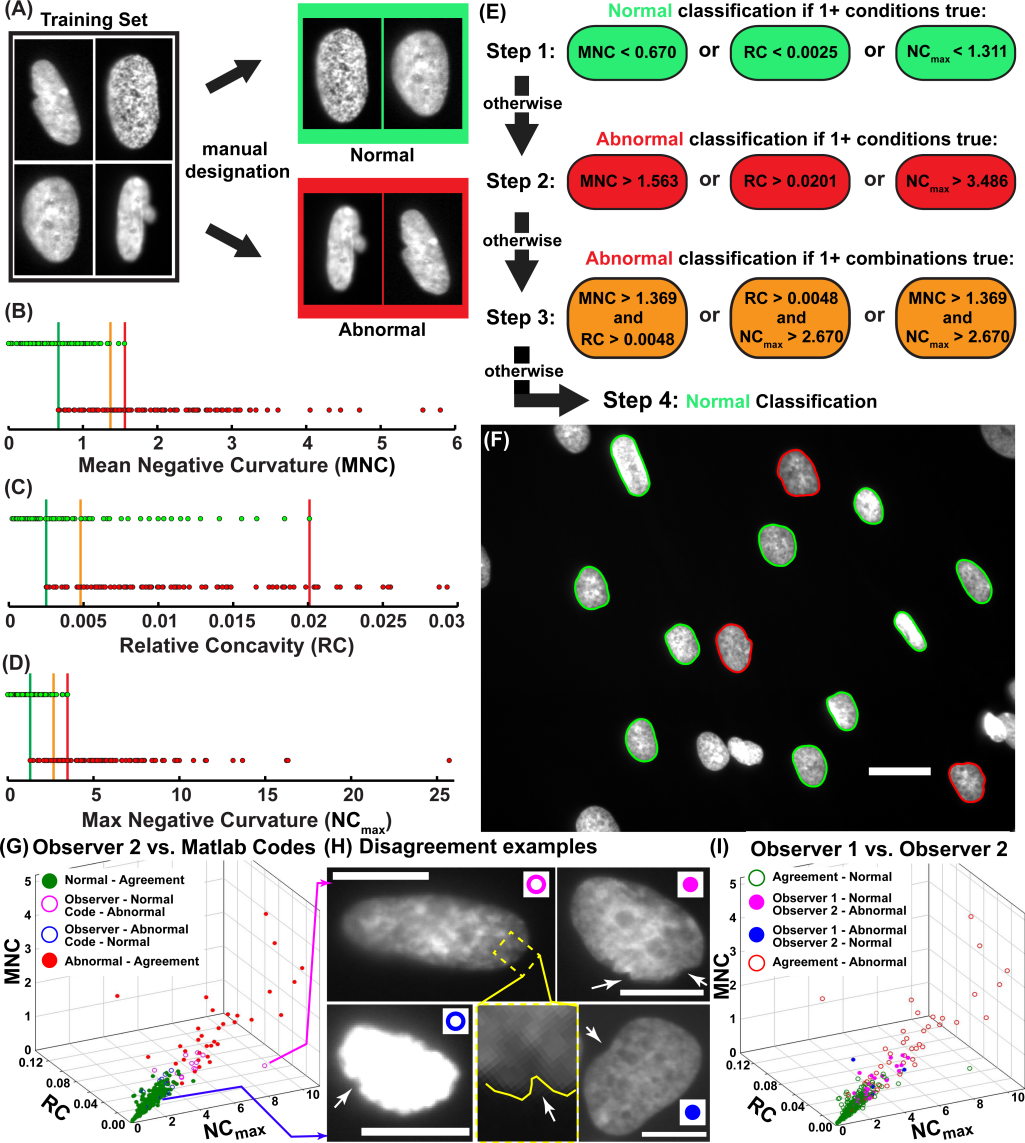
Training, classification scheme, and output. **(**A) Training set nuclei were manually designated by observers as normal or abnormal; Distribution of training set nuclei by (B) mean negative curvature, (C) relative concavity, and (D) maximum negative curvature; (E) Algorithm classification scheme. Step 1: All nuclei below at least one lower limit (shown in green, B-D) are classified as normal. Step 2: All other nuclei above at least one upper limit (shown in red, B-D) are classified as abnormal. Step 3: Nuclei possessing shape feature values between the lower and upper limits but above two intermediate limits (shown in orange, B-D) classified as abnormal. Step 4: Nuclei not satisfying any of these conditions are classified as normal; (F) Automatic classification of nuclei as normally or abnormally shaped based on classification scheme. Scale bar: 25 μm. (G) 3D clustering of normal and defective nuclei for the validation set with Observer 2 vs. Matlab codes; (H) Disagreement examples for Observer 2 vs. Matlab codes, and between two observers; white arrows point to possible defects noticed by an observer; inset highlights a small invagination missed by the observer; Scale bar: 10 μm; (I) 3D plot from G color coded for agreement and disagreement between the two observers.

A training data set was created by cropping 243 LMNA-CM patient and control nuclei from their images using ImageJ (68 U.N.-CONTROL, 101 R.N.-CONTROL, and 74 LMNA-CM). All nuclei in the training set were manually designated by at least two observers as either dysmorphic or normally shaped (Fig 2A). Using the Matlab codes, mean negative curvature (Fig 2B), relative concavity (Fig 2C), and maximum negative curvature (Fig 2D) were calculated for each nucleus in the training set. The manually designated normal nucleus possessing the greatest calculated feature value in the training set became the upper limit for that feature (Red lines in Fig 2B–2D), while the manually designated dysmorphic nucleus possessing the lowest feature value became the lower limit for that feature (Green lines in Fig 2B–2D). In order to differentiate nuclei with feature values between these upper and lower limits, an intermediate limit was also created (Orange lines in Fig 2B–2D). The intermediate limit for each feature was set to a value to minimize the combined number of manually labeled abnormal nuclei below the limit and manually labeled normal nuclei above it to minimize the amount of classification error.

A classification scheme was created using the three limits for each feature. Any nucleus possessing a relative nuclear concavity, mean negative curvature, or maximum negative curvature below the lower limit for that feature was designated as normal (Step 1, Fig 2E). Then, any nucleus above the upper limit for any of the three features was designated as dysmorphic (Step 2, Fig 2E). Additionally, any nucleus in which two of the aforementioned features exceeded the corresponding intermediate limits was also designated as dysmorphic (Step 3, Fig 2E), and all other nuclei were designated as normal (Step 4, Fig 2E). This classification scheme could then be used to automatically designate new nuclei as normal or dysmorphic (Fig 2F). The method was tested against a validation set of nuclei manually classified by two independent observers, and the agreement was found to be above 90% (Table 2). To aid in visualization of disagreements, the three parameters were plotted for each nuclei of the validation set color coded for classification (Fig 2G). The outlier (pink circle with pink arrow pointing to nuclei Fig 2G and 2H) occurred due to the Matlab code independently thresholding each nuclei and outlining a sharp invagination not noticed by the observer during rapid classification. The observer classified an overexposed nucleus as abnormal (Fig 2H, blue open circle nuclei), while the Matlab code draws the outline around gray pixels that are impossible for an observer to notice next to the bright white of the over-exposure. In both of the above cases, it could be argued that the Matlab code suite outperforms the manual observer. Most of the disagreements between the automated Matlab code analysis and the observer occur in the same region as the disagreements between the two observers (Fig 2I). In this region, the defects are slight (Fig 2H) and are easily missed by the observers. Therefore, the consistency of the automatic Matlab codes is an advantage of our method.

**Table 2:** Quantified rating reliability among observers and Matlab codes. Inter-rater reliability between observers and Matlab codes, as well as intra-rater reliability of one observer and the Matlab codes, was quantified using Cohen’s Kappa. Mathematically, the Cohen’s Kappa ranges from zero to one with larger values indicating better consistency. Acceptable values have been debated, but values above 0.6 have generally been considered acceptable [29]. A label of 72 hours. indicates a second trial performed by the same observer or Matlab codes 72 hours following the first. The percent agreement was also calculated for each pair and was consistently above 90%.

Individual and Trial	Cohen’s Kappa	% Agreement
Observer 1 vs. Observer 2	0.59	90.2%
Observer 1 vs. Matlab Codes	0.59	90.0%
Observer 2 vs. Matlab Codes	0.71	94.4%
Observer 1 vs. Observer 1 (72 hrs.)	0.70	91.3%
Matlab Codes vs Matlab Codes (72 hrs.)	1.00	100%

### Effect of lamin A/C staining on nuclear shape analysis

To demonstrate the method’s versatility, it was used to analyze images of nuclei labeled by different fluorescent stains. When the same nuclei were visualized using both Anti-Lamin A/C Antibody (S3A Fig) and DAPI (S3B Fig), no significant differences were observed in area, eccentricity, and mean negative curvature calculated by the Matlab codes (S3C Fig). The percentage of nuclei automatically designated as dysmorphic between the two image sets were also not significantly different (S3F Fig).

### Characterizations of nuclear morphology

**Fig 3:**
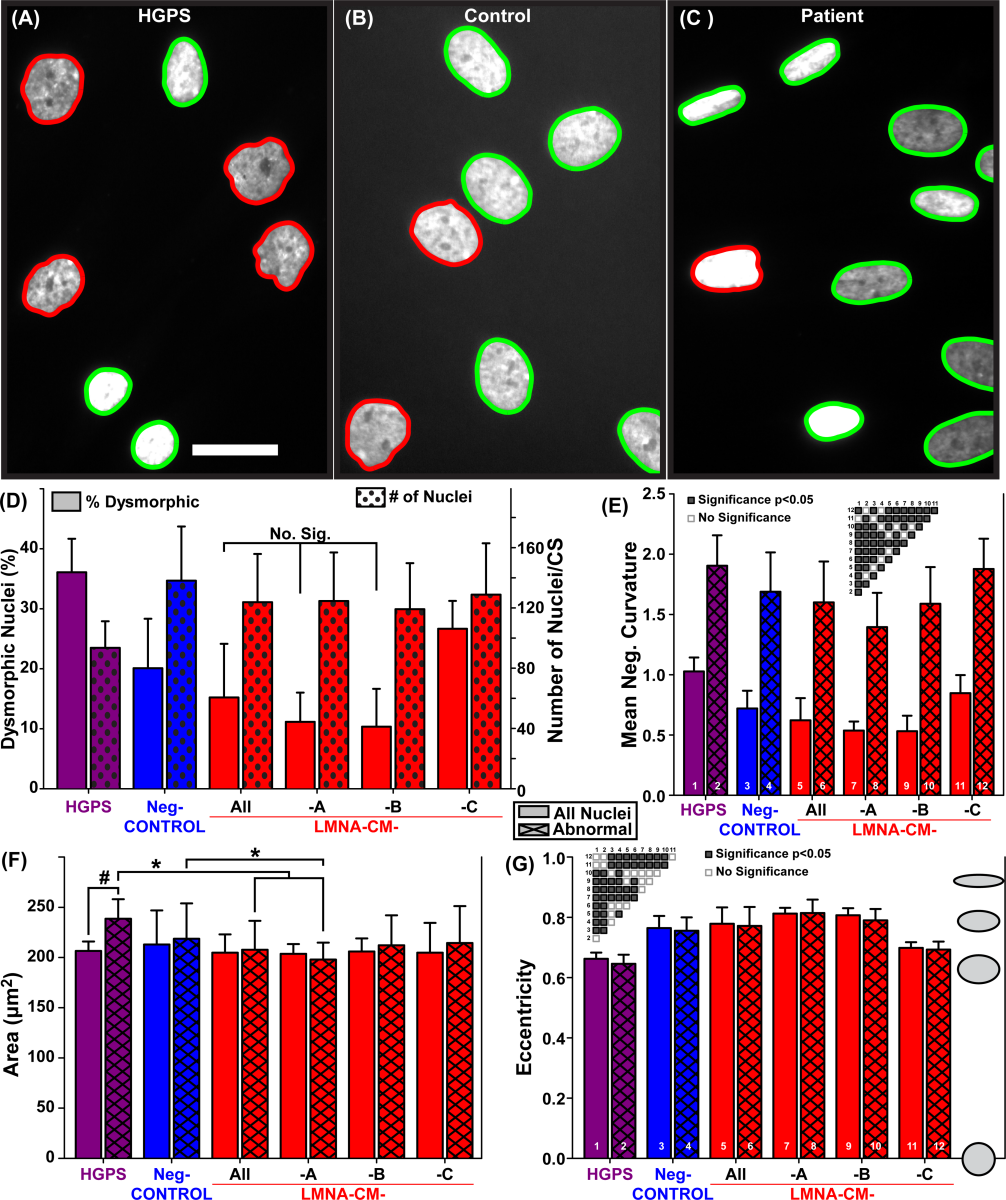
Classification and nuclear morphology measurements in skin fibroblast nuclei. Examples images for: (A) HGPS positive control nuclei, (B) R.N.-CONTROL- Family A, and **(**C) LMNA*-*CM-A nuclei. Nuclei outlined in green have been designated as normal, while nuclei outlined in red have been designated as dysmorphic; (D) Percentage of dysmorphic nuclei and number of nuclei per coverslip; For % dysmorphic, significance was found for all pairs except for the ones indicated. For the number of nuclei, only HGPS was significantly different. **(**E) Mean negative curvature; significance is indicated with a matrix on the plot. (F) Nuclear area; # denotes significance in an internal pair comparison between overall and dysmorphic condition with the t-test; * denotes significance found among conditions with the ANOVA test. (G) Nuclear eccentricity with the significance among groups indicated in the matrix. Error bars: one standard deviation. Sample sizes for the HGPS, Neg-CONTROL, LMNA-CM, LMNA-CM-A, LMNA-CM-B and LMNA-CM-Care 11, 101, 75, 27, 27, and 21 respectively and the passage numbers for all the samples are indicated in Table 1. Scale bar: 25 μm.

The nuclei from an HGPS (Fig 3A), Neg-CONTROL (Fig 3B), and LMNA-CM (Fig 3C) conditions were analyzed, and average nuclear area, eccentricity, and mean negative curvature were calculated for all groups including a breakdown by family. The averages among nuclei automatically designated as dysmorphic were also calculated for each group.

Consistent with morphological observations of HGPS cell nuclei, the average percentage of dysmorphic nuclei was significantly greater in HGPS patient coverslips than in those of all negative control individuals (Fig 3D). Surprisingly, the average percentage of dysmorphic nuclei in LMNA-CM group was significantly lower than in the Neg-CONTROL group despite a similar average age between the groups. Additionally, the average mean negative curvature of dysmorphic nuclei for some of the families was significantly reduced compared to their HGPS positive control and Neg-CONTROL counterparts, which did not differ significantly from each other (Fig 3E).

The average area of all HGPS nuclei was significantly lower than that of dysmorphic HGPS nuclei alone (Fig 3F). No significant difference in area was observed between dysmorphic nuclei and overall nuclei in the other groups, but the area of the LMNA-CM-A dysmorphic nuclei was significantly smaller than in either the positive (HGPS) or negative controls (Neg-CONTROL) groups. The area of the defective nuclei for each individual was also plotted as a function of age showing no apparent correlations (S5 Fig). The average eccentricities of HGPS nuclei were significantly lower than that of Neg-CONTROLs, while some of the LMNA-CM- families (A & B) had nuclei eccentricity that was significantly higher than that of negative controls (Fig 3G).

### Correlation with onset of clinical symptoms

**Fig 4:**
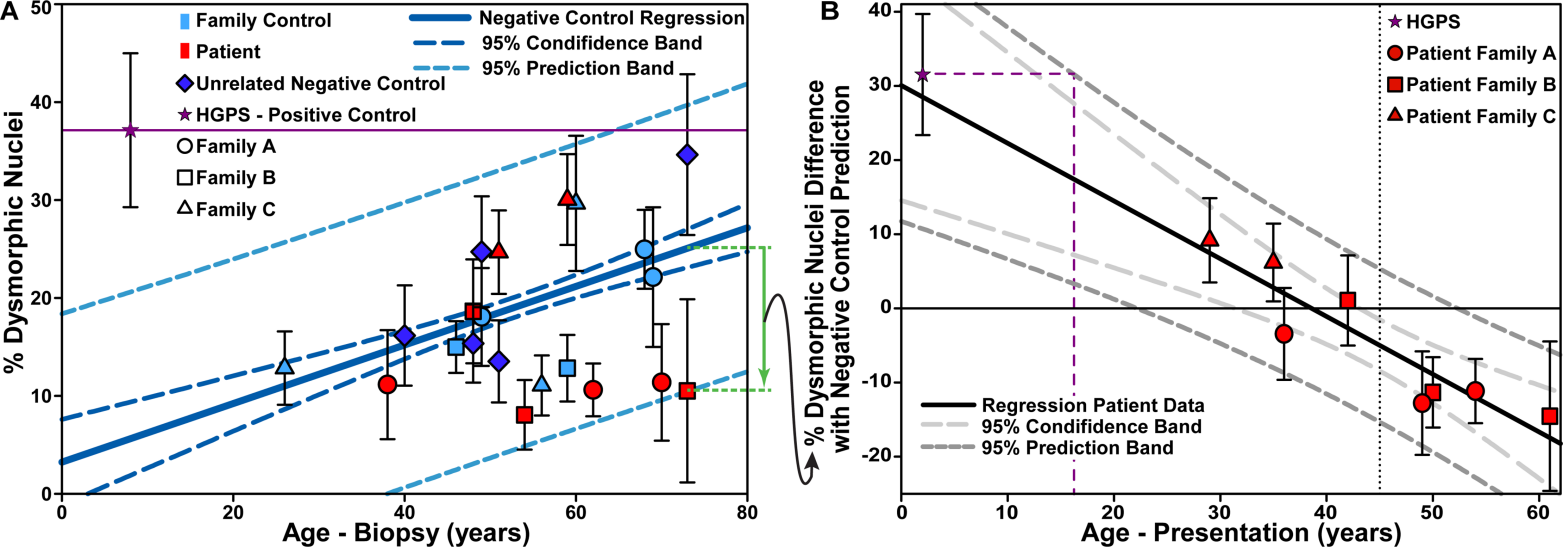
Nuclear defects and age. (A) Percentage of dysmorphic skin fibroblast nuclei in LMNA*-*CM patients, related and unrelated negative controls, and HGPS patient, versus age of biopsy. Regression line (solid dark green line), confidence and prediction intervals (long dark green dashes and short light green dashes, respectively) correspond to data of all related and unrelated negative controls (R^2^ = 0.21, P_slope_ < 0.001). Purple line serves as a visual guide of the percentage of dysmorphic skin fibroblast nuclei in the HGPS patient. Blue line, as an example, displays difference of dysmorphic nuclei percentage of a patient and the control regression line. Each individual (plotted point) represents an average from 4–12 samples, with the average sample size n = 8.5; (B) At age of biopsy for an individual, the difference between dysmorphic nuclei is plotted against age of symptom onset. Regression line (solid black line), confidence and prediction intervals (long light gray dashes and short dark gray dashes, respectively) has an R^2^ = 0.84 and P < 0.003. In addition, HGPS data collapses on the master curve of the linear regression trend. Passage number for all the samples are indicated in Table 1.

In Fig 4A, the percentage of dysmorphic nuclei in the cells from people with no mutations (unrelated controls-dark blue, related controls-light blue) was plotted against the age at which the biopsy was taken. As expected [31], the percent of dysmorphic nuclei increases with age of biopsy for negative controls (Fig 4A, blue solid line). It was found that the patient data for all three families fell within the 95% prediction band of the control trend line.

Unlike HGPS patients (purple star Fig 4A), the patients in the three families (LMNA-CM) do not exhibit skin abnormalities, and thus it is unsurprising that only the HGPS samples lie outside the prediction interval. However, majority of the LMNA-CM individuals are below the trend-line, which explains the aggregated average percent defective for LMNA-CM and LMNA-CM-A groups. From clinical data [4,9,10], we knew that the older patients in the cohort (LMNA-CM) developed heart disease later in life. Therefore, we plotted the difference between each patient’s cells’ dysmorphic nuclei percentage and the predicted value based on the control trend line (green arrow, Fig 4A) against the age at which patients first presented with heart disease symptoms (Fig 4B). Patients with higher positive deviation from negative controls’ trend-line were found to present with heart disease symptoms at a younger age (prior to 45 years, vertical dotted line Fig 4B). Indeed, there is a significant linear correlation (R^2^ = 0.84, P < 0.003) between patient separation from age-matched negative control prediction and the onset of symptoms age. While the Coriell Institute does not provide the information of when this particular patient (catalog # AG11513) developed heart disease symptoms, it is typical for individuals with the HGPS disease to present with heart disease symptoms very early in life [32,33]. To compare the HGPS positive control to the LMNA-CM group, it was similarly plotted assuming a presentation age at 2 years (purple star—Fig 4B). Interestingly, the normalized data for the HGPS cell-line will fall within the prediction limit of the patient trend-line (black line—Fig 4B) as long as heart disease symptoms were first developed in this particular patient prior to 16 years of age, which is very likely based on clinical reports [32,33].

## Discussion

The automatic quantification and characterization of nuclei proposed in this work was validated by comparing the negative and positive control cell lines. For example, both nuclear area and eccentricity were consistent with previously described trends between HGPS and negative control fibroblast nuclei [34–36]. The automatic classification and counting of dysmorphic nuclei provides advantages over the exclusive use of averaged shape features such as mean negative curvature. Measuring the proportion of dysmorphic nuclei present in a tissue is in many cases a more intuitive metric to compare the prevalence of defective nuclei in cells from specific tissues and individuals. Furthermore, the individual identification of dysmorphic nuclei allows researchers to specifically examine the morphology of those nuclei. Dermal fibroblasts cultured from most individuals, even those with pathologies which cause severe nuclear blebbing such as HGPS, possess a mixture of both dysmorphic and normally shaped nuclei [37]. Shape descriptors such as mean negative curvature are unable to provide information pertaining to a subset of these populations, always incorporating the measured values of normal nuclei into a calculated average. Additionally, our method demonstrated that unlike LMNA-CM patient and control nuclei, dysmorphic and normal HGPS nuclei possessed a significant area difference. While differences in average area compared to negative controls and an increase in average area by late passages in HGPS nuclei have been previously described, the size difference between the normal and dysmorphic nuclei present in the same HGPS cell population prior to this work were either qualitatively observed or not interrogated [34,37,38]. One explanation put forth for the prevalence of giant nuclei in HGPS cell populations is mitotic abnormalities leading to polyploidy [39]. By providing quantitative data regarding the relative size of dysmorphic HGPS nuclei, our Matlab codes may aid in substantiating such hypotheses in the future. Unlike previous methods applied to nuclear shape analysis [20,28,40,41], we are able to determine whether alterations to nuclear morphology other than a misshapen envelope in diseased cell lines, such as changes in area, apply solely to dysmorphic nuclei or to the entire population (Fig 3F).

Automatic classification of images has been extensively studied. Matlab software has substantial image analysis capabilities and open source sub-routines used in our method [42–45]. Similar open sourced software, CellProfiler, has also been utilized to analyze images [46–48]. Indeed, CellProfiler in combination with Matlab has been used to analyze the morphology of the nuclei in multiple cell types to determine the mechanisms of Cofilin [48], but the actual CellProfiler and Matlab codes are not provided. The Matlab codes we included in the supporting information (S1–S7 Codes) have already been trained for the described task and provide a variety of unique outputs (Fig 3) not readily available from basic CellProfiler or Matlab installations. Another method for automated classification would involve machine learning such as neural networks [25,49–52]. This method is more complex than what was utilized in our software codes, but it can be more powerful for classifying subtle differences. For example, classifying cancerous breast tissue manually requires pathologists to be trained for multiple years. In such a case, machine learning is essential to capture the multitude of image properties analyzed by an experience human eye [52]. Thus in a simpler classification problem, such as determining defective nuclei from a fluorescent stain image, there was no need to employ the more complex methods of machine learning.

The capability of our simpler automated method was demonstrated by applying it to characterize fibroblast nuclei from *LMNA* mutation patients who mainly present with heart disease. The significant difference in nuclear area (overall and defective) between LMNA-CM-A and both the positive and negative controls (Fig 3F) was not driven by one individual (S5 Fig), but indicates an overall trend. Along with the significantly higher eccentricity of the LMNA-CM-A and LMNA-CM-B nuclei (Fig 3G), this indicates potential avenues of investigation in terms of the elastic properties of the nuclei lamina caused by specific mutations. Indeed, the larger average eccentricity observed in LMNA-CM-A and LMNA-CM-B nuclei compared to either HGPS or controls could indicate greater deformability, a quality with potential relevance in diseases such as cardiomyopathies, which affects cyclically contracting and mechanically strained tissues. Conversely, the significant reduction in the average percentage of dysmorphic nuclei in the LMNA-CM compared to the negative controls (Fig 3D) is driven by a few individuals who have low numbers of defective nuclei for their age group (Fig 4A). For example, the individual from Family A who exhibited an onset of heart disease symptoms at age 36 had approximately the same number of nuclei defects as an older patient in Family A who did not exhibit heart disease symptoms until age 56 (Fig 4). In general, those below and above the trend-line in Fig 4A can be viewed as aging “slower” and “faster” than the average population, respectively. Thus, while the amount of defective nuclei in the LMNA-CM patients fell within the prediction interval of a negative control population, the “slow aging” individuals seemed to be partially protected from the effects of the mutation and, consequently, developed heart disease later in life (Fig 4B). This difference between patients is likely driven by genetic modifiers [44] and based on our data, we plan to search for proactive genetic modifiers in Family A. These results also help explain the surprisingly low average percent of dysmorphic nuclei in the LMNA-CM population (Fig 3D). Indeed, the LMNA-CM population is skewed by the lack of “faster aging” elderly patients, which, we believe, is the results of these individuals developing heart disease early in life with fatal consequences.

By individually identifying and characterizing dysmorphic nuclei, our method provides an opportunity to explore subtle differences among individuals and populations. As the method was automated, it was possible to analyze cell-lines from 22 individuals (over 25 thousand nuclei in total) spanning four different *LMNA* mutations and appropriate age-matched controls. The results provide further avenues of investigation of the genetic modifiers protecting some patients as well as the mechanisms linking the mutation to the disease. This powerful method can also, in the future, be applied to any other cell type or disease where investigating nuclear morphology could elucidate pathological mechanisms.

## Supporting information

S1 Text Detailed description of analysis method and matlab codes. (PDF)

S1 Code NucleiDetect.m is a matlab code that outlines the nuclei in the images. The detailed description of the code is provided in S1 Text.
(M) Click here for additional data file.

S2 Code NucleiClassify.m is a matlab code that classifies the nuclei as normal or defective. The detailed description of the code is provided in S1 Text.
(M) Click here for additional data file.

S3 Code BoundaryRefine.m is a matlab function used by NucleiDetect.m. The detailed description of the code is provided in S1 Text.
(M) Click here for additional data file.

S4 Code calc_circle.m is a matlab function used by NucleiDetect.m. The detailed description of the code is provided in S1 Text.
(M) Click here for additional data file.

S5 Code curvature.m is a matlab function used by NucleiDetect.m. The detailed description of the code is provided in S1 Text.
(M) Click here for additional data file.

S6 Code curvature_circle.m is a matlab function used by NucleiDetect.m. The detailed description of the code is provided in S1 Text.
(M) Click here for additional data file.

S7 Code ColoredNuclei.m is a matlab function used by NucleiClassify.m. The detailed description of the code is provided in S1 Text.
(M) Click here for additional data file.

S1 Fig Overview of image analysis. Starting with an unprocessed, 2D fluorescent nuclei image and ending with the generation of nuclei boundaries, which are overlaid on the original image. Boxes represent steps in the image analysis process while arrows represent progression to a new or previous step.
(TIF)

S2 Fig Process for manual exclusion of overlapping nuclei. **a** Following automatic segmentation of nuclei, an image is displayed with all detected nuclei highlighted and numbered, including one undetected pair of overlapping nuclei; **b** User is prompted to choose an action after reviewing the displayed image, entering ‘6’ in order to exclude the overlapping nuclei from further analysis, and then entering the number corresponding to that body in the image; **c** The overlapping nuclei are excluded from analysis, and highlighted in red.
(PDF)

S3 Fig Consistency of nuclear morphology measurements of Matlab codes across multiple fluorescent stains. Nuclei were visualized with both **a** Lamin A+C immunofluorescence label and **b** DAPI, and then segmented by the image analysis Matlab code; **c** Average area, **d** average eccentricity, and **e** average mean negative curvature were then calculated based on nuclear boundaries in both the DAPI and Lamin A+C stain images (n = 48 nuclei). **f** The percentage of nuclei designated as dysmorphic was also calculated for all images and averaged by stain type (n = 12 images). Error bars represent one standard deviation. Scale bar: 25 μm.
(TIF)

S4 Fig Image processing steps for nuclear boundary extraction. **a** Original grayscale image; **b** Binary image generated from first intensity threshold; **c** Regions of original grayscale image isolated based on detected bodies in binarized image; **d** Region of original image corresponding to a single nucleus; **e** Binary image generated from second intensity threshold; **f** Outline of nucleus generated; **g** Outline smoothed and given sub-pixel resolution using snake algorithm; **h** Nuclei labeled and outlined with snake algorithm boundaries; **i** Overlapping nuclei (red outline) excluded from analysis; **j** Example data of nuclear area and orientation. Scale bar: 25 μm.
(PDF)

S5 Fig Plot of defective nuclei area as a function of the age at which biopsy was taken for each individual. Plot shows no correlations for the data.
(PDF)
