# Supplementary material for: Age of heart disease presentation and dysmorphic nuclei in patients with LMNA mutations
Source: PLoS One. 2017 Nov 17;12(11):e0188256. doi: 10.1371/journal.pone.0188256 (PMC5693421; doi:10.1371/journal.pone.0188256)
Supplement: S1 Text — (PDF) [file pone.0188256.s001.pdf]

Supplementary Text S1

Nuclear Boundary Extraction using *NucleiDetect*

In order to facilitate segmentation of nuclei from the image background, pixel intensity values within an image were first adjusted using a contrast enhancement function (*imadjust* in the MATLAB Image Processing Toolbox). MATLAB's automatic thresholding function (*im2bw*), which utilizes Otsu's method (Otsu, 1979), then determined a global intensity threshold value. For each image, this global threshold was multiplied by a user-input value to optimize the binarization process and minimize background noise and errors in nucleus segmentation. Applying this final threshold value converted each nucleus into a body of white pixels among a black background, with black holes in each white body being filled (*imfill*) (Fig. S4B). Thereafter, an initial boundary for each body was traced (*bwboundaries*), and any bright region with a perimeter of less than 8 microns was excluded as noise. Next, only the bright regions entirely encompassed within the image and not in contact with its edge were included in further analysis (highlighted by red rectangles in Fig. S4C).

Smaller images extending 15 pixels past the highest, lowest, right-most and left-most boundary points of each nucleus (dimensions shown by red rectangles in Fig. S4C) were automatically cropped from the original image (Fig. S4D), and then subjected to a second round of pixel intensity adjustment using *imadjust*. Because background pixel brightness can vary considerably within a single image, limiting pixel contrast enhancement to each nucleus and the area immediately surrounding it enabled superior segmentation and smoother boundary extraction, often eliminating outline noise in cases of slight overexposure or varying background contrast. The binarization by the same threshold (*im2bw)* (Fig. S4E) and boundary tracing process (*bwboundaries*) (Fig. S4F) was then repeated on each new image of a single nucleus.

Due to their pixel-level resolution, these boundaries still possessed small amounts of noise which, if not reduced, would create greater difficulties in automatically differentiating between normal and deformed nuclei (Fig. S4G). To address this, the outer convex hull of the

newly traced nuclei boundaries were calculated (*convhull*), and then used to initiate the active contour algorithm devised by Xu and Prince (*Snake2D*) [18]. The natural coordinates of each nuclear boundary were also interpolated to consist of 1000 evenly-spaced points using a function included in the snake algorithm library (*InterpolateContourPoints2D*). The final nucleus outlines calculated by this algorithm possessed both reduced noise and sub-pixel resolution (Fig. S4G) for all fully visible nuclei (Fig. S4H). Some outlines corresponded to multiple nuclei either in contact with each other or overlapping, preventing extraction of their entire boundaries and thus a complete characterization of their shape (Sup. Fig S2). The user was prompted to manually identify these and exclude them from further analysis (Fig. S4J & Sup. Fig S2).

The third-party MATLAB codes used to calculate areas of nuclear boundaries, fit ellipses to nuclear boundaries to enable calculation of eccentricity, and perform boundary smoothing using the Snake algorithm are available on the MATLAB Central File Exchange (Chernov, 2009; Kroon, 2010; Sommer, 1998). Alternatively, we provide executables of NucleiDetect and NucleiClassify, which will run without the need for downloading the codes.

Validation of Automatic Nuclei Classification

A validation set consisting of 449 nuclei *LMNA*-CM patient and control nuclei across 20 images was then used to determine the level of agreement of classification between the codes and two independent, manual observers, and the error between the observers themselves. Inter-rater reliability and intra-rater reliability were both measured using the Cohen's kappa statistic. The calculated Cohen's kappa values between manual observers, between each observer and the codes, and between the same observer in two classification trials 72 hours apart, were all comparable. As expected, the codes exhibited perfect intra-rater reliability through complete agreement between two classification trials 72 hours apart, an improvement over the intra-rater reliability seen in manual observation (Table 1).

Analysis Codes

Below are step-by-step instructions on how to analyze fluorescence images of DAPI-stained nuclei using *Nucleidetect*.

1a. Organize all MATLAB codes or executables

a. All MATLAB codes are intended to run on MATLAB version 2015b.

b. MATLAB codes needed to run *NucleiDetect* and *NucleiClassify* should all be located in the same folder

c. All necessary MATLAB codes are as listed:

**BoundaryRefine.m**
**calc_circle.m**
**ColoredNuclei.m**
**curvature.m**
**curvature_circle.m**
**DrawDegmentedArea2D.m***
**ellipsefit.m***
**ExternalForceImage2D.m***
**GetContourNormals2D.m***
**GVFOptimizeImageForces.m***
**ImageDerivatives2D.m***
**imgaussian.m***
**InterpolateContourPoints2D.m***
**MakeContourClockwise2D.m***
**NucleiClassify.m**
**NucleiDetect.m**
**polygeom.m***
**rad2deg.m**
**Snake2D.m***
**SnakeInternalForceMatrix2D.m***
**SnakeMoveIteration2D.m***

d. NOTE: All codes marked by an * are third party and must be downloaded from Central File Exchange. Links provided in references and below:

polygeom:

www.mathworks.com/matlabcentral/fileexchange/319-polygeom-m

ellipsefit:

www.mathworks.com/matlabcentral/fileexchange/22684-ellipse-fit--direct-method

Snake2D and associated functions:

www.mathworks.com/matlabcentral/fileexchange/28149-snake---active-contour

1b. Standalone executable versions of code

a. As an alternative to the original MATLAB codes, users may download and run NucleiDetect and NucleiClassify as executable programs.

These are available at the following google drive link: https://drive.google.com/drive/folders/0B4lF4nn4ku9fMkZsSnlUc08tbGc?usp=sharing

i. The applications are located within the 'for_redistribution_files_only' folders:

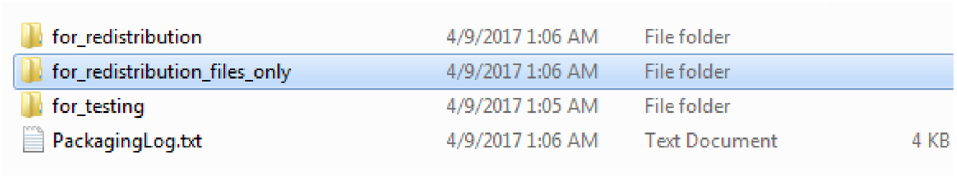

b. If you receive the following error:

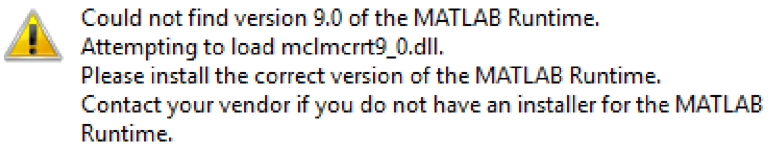

Make sure you have installed MATLAB Compiler Runtime 9.0. This can be accomplished by running the 'MyAppInstaller_web.exe' application provided in the 'for_redistribution' sub-folder:

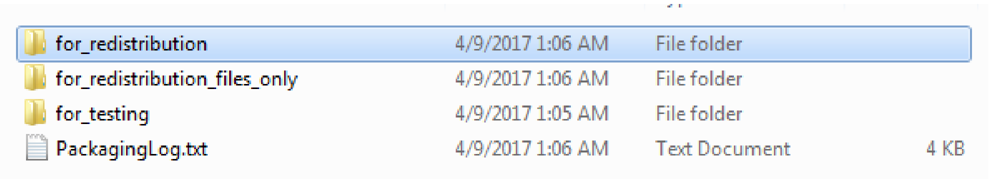

c. If you receive the following error:

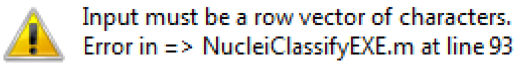

You have selected 'cancel' in the dialogue box or not entered any input after selecting 'Ok'. The application will run properly if recommended input is provided.

2. Organize image files

a. Image files to corresponding to the same coverslip should all be located in the same folder

b. Image file names should be consistent and correspond to their condition and coverslip.

c. Images to be analyzed should be of sufficiently good quality:

i. Nuclei should be in focus

ii. Nuclei must not be excessively overexposed or underexposed

iii. Tissue should not be over-confluent

Acceptable Image

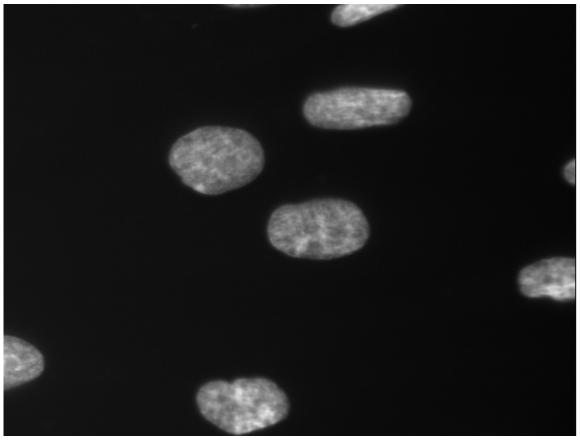

Nuclei not in focus

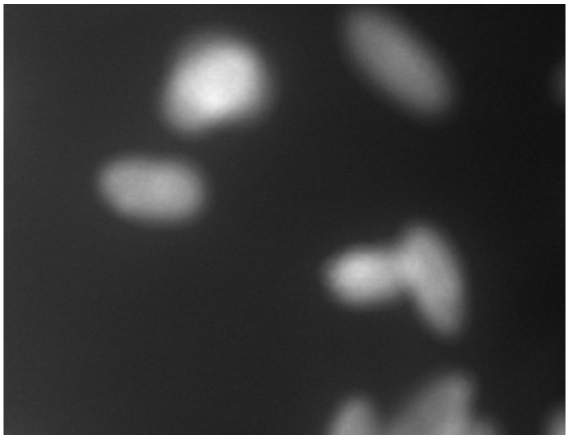

Nuclei underexposed/overexposed

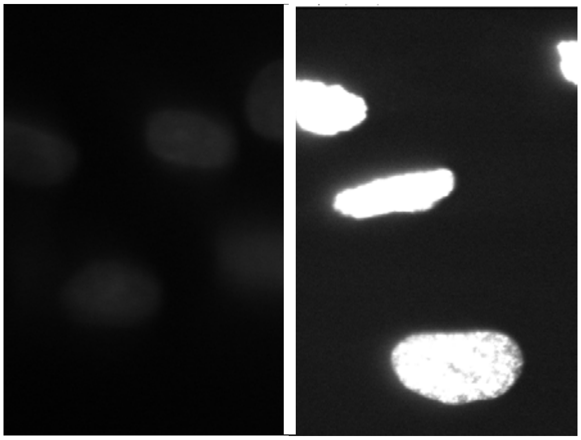

Over-confluent

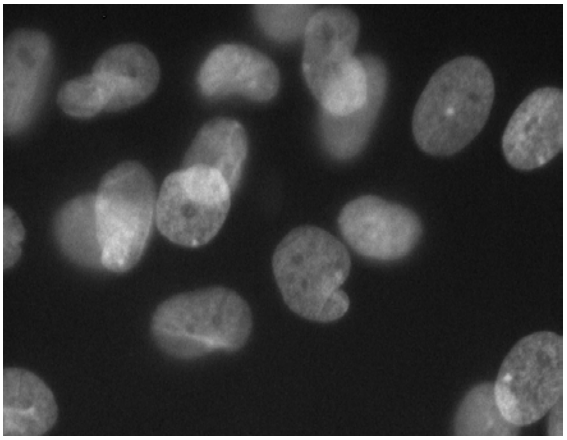

3. Set image directory in Nucleidetect

a. You may change the directory in which Nucleidetect searches for the image files for your convenience (outlined in red).

```
pathfiles = 'Z:\2016-01-21_Z\Patient_A1_p7_ISO_WELL1';
% Ask user to select files
[file,path]=uigetfile({'*.TIF';'*.*'},'Select Images...', pathfiles,'MultiSelect','on');
```

b. If running the executable version, you will be prompted by a dialogue box to enter the path:

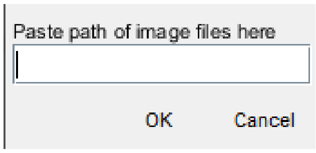

4. Set pixel-to-micron conversion value in Nucleidetect

a. This value is saved in output files and does not need to be re-specified for *NucleiClassify*

```
% Conversion factor between image pixels and microns. Equals number
% of pixels in one micron
conversion_factor = 6.24;
```

b. In the executable version, you will be prompted for the value in a dialogue box

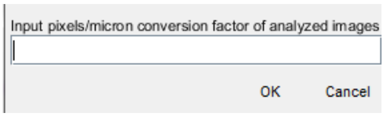

5. Run Nucleidetect

a. Choose whether to save Nucleidetect outputs in command prompt

```
>> NucleiDetect_2017_01_25
Save Outputs? 0 = Yes 1 = No
```

b. Select all images to analyze in the prompt.

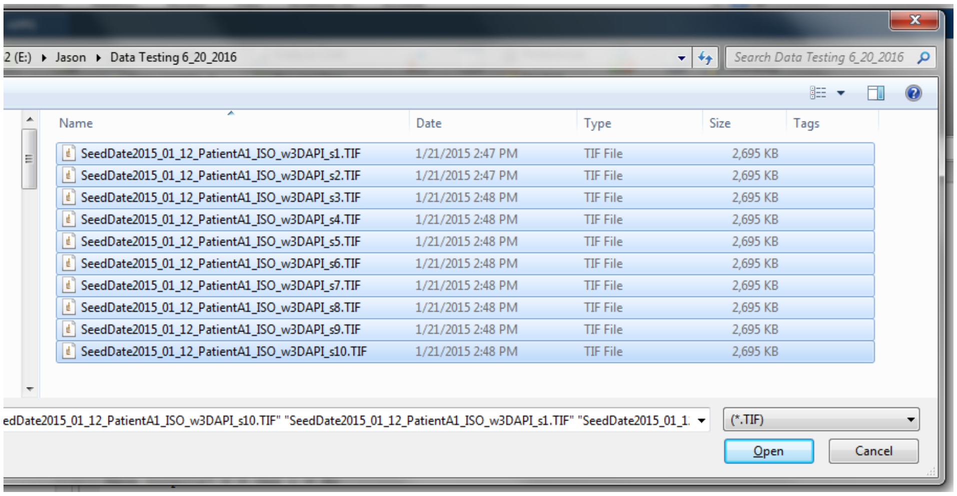

c. Choose starting threshold. Recommended values suggested in command line.

i. The threshold controls detection of nuclei based on pixel intensity.

ii. Larger threshold: less of image will be converted to white and registered as belonging to a nucleus

iii. Smaller threshold: more of image will be converted to white and registered as belonging to a nucleus

d. You will then be prompted to enter a number based on which action you wish to take:

```
Accept Values? (yes = 0, no = 1, throw out objects = 6,
 analyze with unique threshold = 8, throw out image = 13, reset = 22):
```

e. Examine traced nuclei boundaries and binarized nuclei image

Threshold too small **Threshold too large**

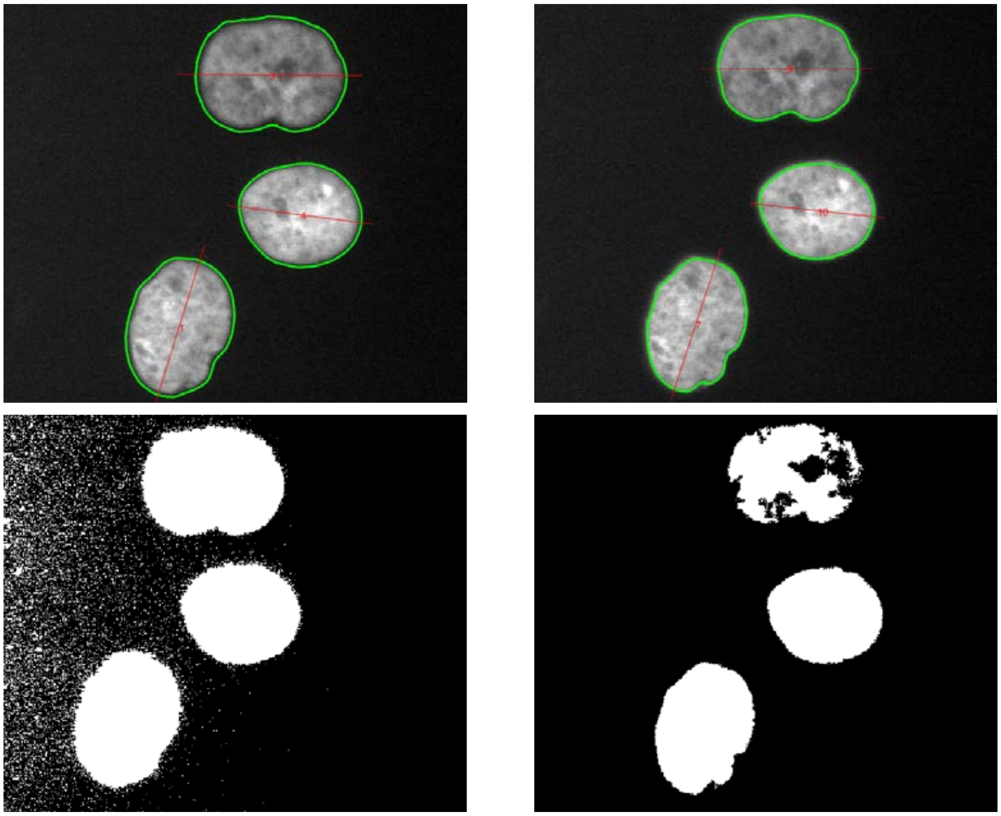

f. Input different threshold if needed, entering '1' in command prompt to enter a new value

i. Once the binarized nuclei bodies and nuclei outlines match the original nuclei in shape, accept the threshold and continue

g. Examine image for undetected overlapping nuclei

i. Some nuclei may be automatically recognized as overlapping and excluded from analysis, highlighted by a red boundary. This is controlled by the overlap detection value (recommended values also displayed in command line)

ii. The default overlap detection value is displayed along with the current threshold value in the command line when asked if image analysis is satisfactory

h. Input different overlap detection value if needed, enter ‘2’ in command prompt to enter a new value

i. To increase the number of nuclei designated as overlapping in the case of false negatives, input a smaller overlap detection value in the same manner as a new threshold input.

ii. To decrease the number of nuclei designated as overlapping in the case of false positives, input a larger overlap detection value in the same manner as a new threshold input

Overlap constant too small

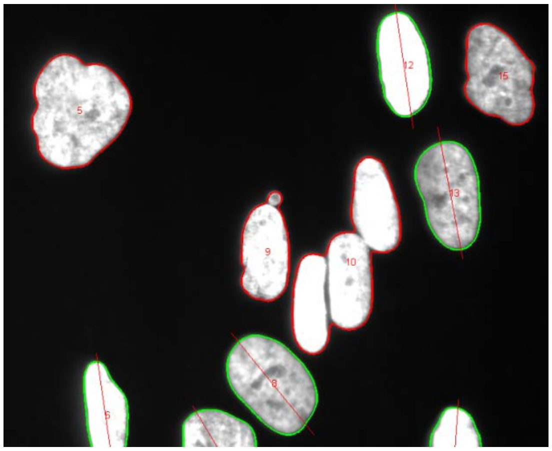

Overlap constant too large

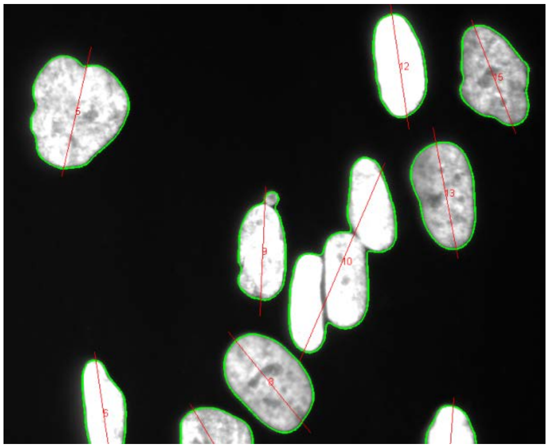

iii. To manually designate nuclei as overlapping, enter ‘6’ when prompted to continue, and then input the number displayed in the center of the overlapped nuclei you wish to exclude from analysis.

iv. Automatic detection of overlapping nuclei can essentially be disabled by entering a sufficiently large overlap detection value.

i. To reset threshold and overlap detection values back to defaults and remove any manual exclusions of nuclei for an image, enter ‘22’ in the command prompt.

i. Accept image analysis when satisfactory

i. To accept image analysis, enter ‘0’ in command prompt

ii. If image analysis cannot be made satisfactory, enter ‘13’ in command prompt to exclude entire image from further analysis and refrain from saving any outputs or data related to the image.

6. <u>Check Outputs</u>

a. If code was directed to save outputs, check outputs in folder containing the original analyzed images

i. The folder should contain an “IMAGE NAME_final image.tif” file for each analyzed image

ii. The folder should contain an “IMAGE NAME_area_eccentricity_perimeter.mat” file for each analyzed image

iii. The folder should contain an “IMAGE NAME_voronoi_input.mat” file for each analyzed image

iv. The folder should contain one “summary.mat” file

7. Set image directory in NucleiClassify

```
pathfiles = 'Z:\2016-03-17_Z\Patient_A1_p7_ISO_Well5';
```

8. Run NucleiClassify

a. NucleiClassify requires that images have been analyzed by Nucleidetect first, and the appropriate output files generated.

b. Determine type of output image to be created.

Colored outlines Colored interiors

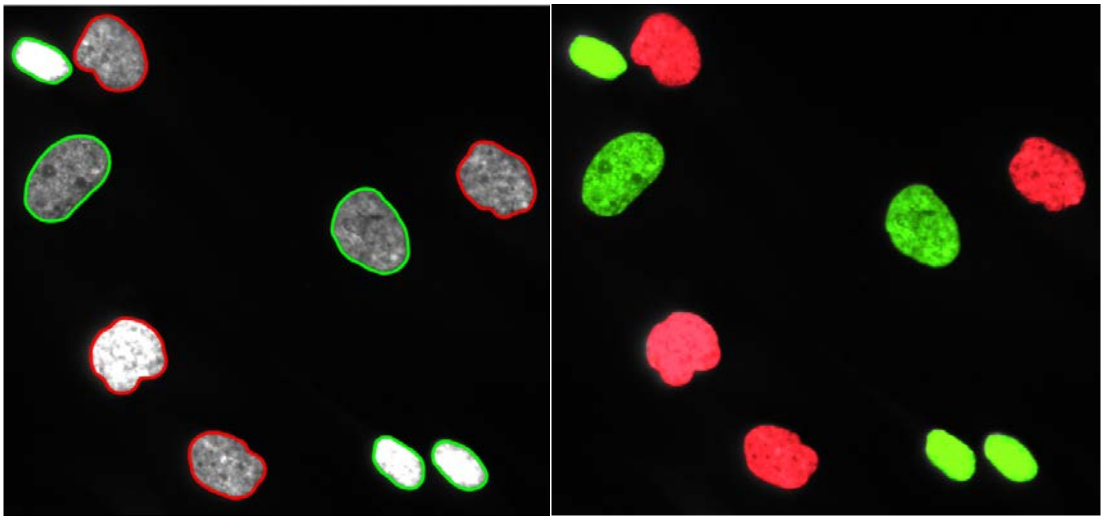

c. Select all “IMAGE NAME_area_eccentricity_perimeter.mat” files corresponding to the images you wish to analyze

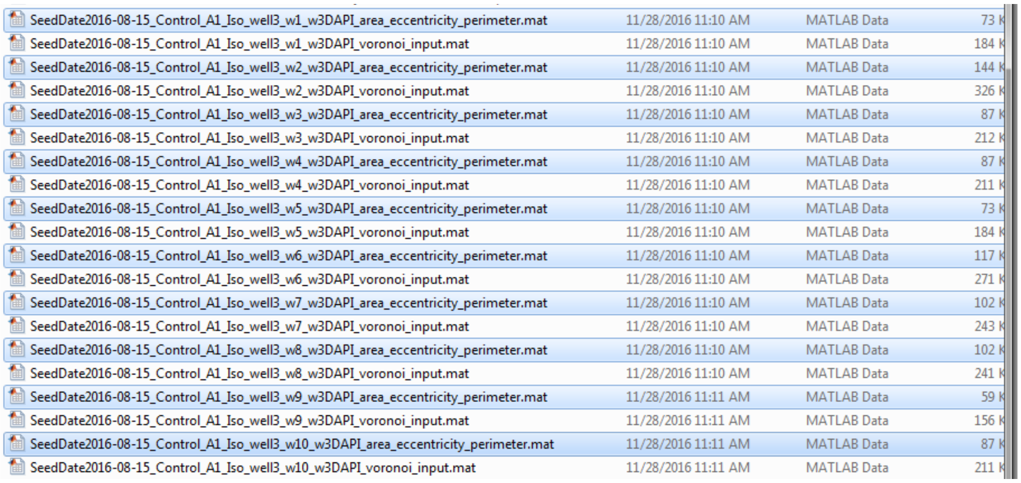

d. Select all images you wish to analyze

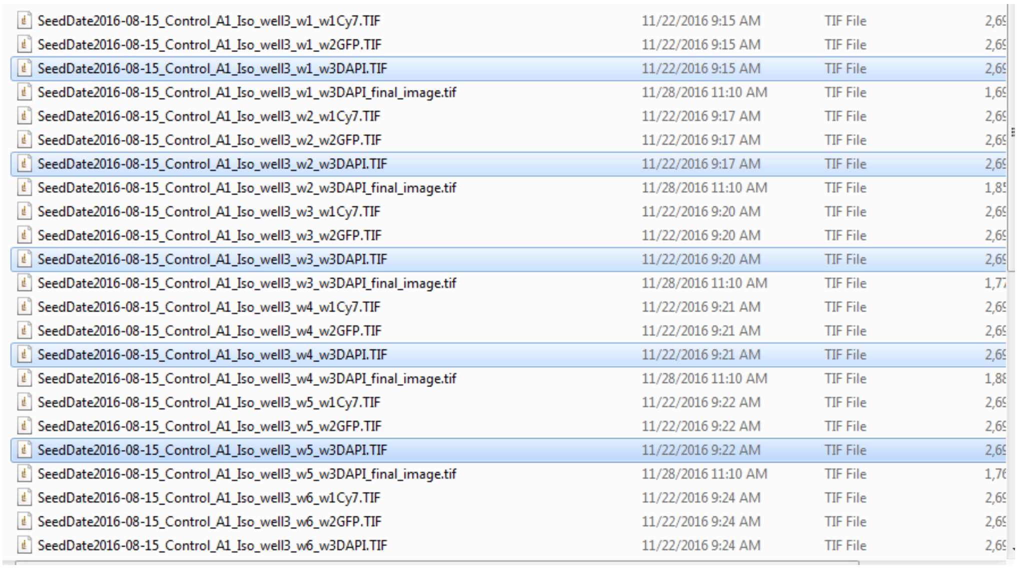

e. Select format of output images (whether nuclei classification is indicated by outline color or whole nuclei coloring)

9. <u>Check Outputs</u>

a. The folder should contain an "IMAGE NAME_area_eccentricity_perimeter_auto_count.tif" file for each analyzed image.

b. The folder should contain one "summary_normal_defectives.mat" file.

Summary of Code Outputs

IMAGENAME_area_eccentricity_perimeter:

B_new	nx1 cell array containing boundaries of each detected nucleus in an image
areas	1xn matrix containing calculated areas (in sq. microns) of each detected nucleus in an image.
eccentricities	1xn matrix containing calculated eccentricities of each detected nucleus in an image
perimeters	1xn matrix containing calculated boundary perimeters (in microns) of each detected nucleus in an image
nonzero_orientation_angles_fov	nx1 matrix containing orientation values of each detected nucleus in an image, defined as the angle of the major axis of the least-squares ellipse fitted to each nucleus
level2	The intensity threshold value set for analysis of an image
throwout	Indicates whether the image has been designated by a user as unfit for analysis
conversion_factor	Number of pixels to a micron in microscope images being analyzed

Summary.mat

areas_total	1xn matrix containing calculated areas (in sq. microns) of each detected nucleus in an analyzed image set.
eccentricities_total	1xn matrix containing calculated eccentricities of each detected nucleus in an analyzed image set.
perimeters_total	1xn matrix containing calculated perimeters (in microns) of each detected nucleus in an analyzed image set.
avg_areas_coverslip	Average calculated area (in sq. microns) of detected nuclei in an analyzed image set.
avg_eccentricities_coverslip	Average calculated eccentricity of detected nuclei in an analyzed image set.
avg_perimeters_coverslip	Average calculated perimeter (in microns) of detected nuclei in an analyzed image set.
nonzero_orientation_angles_coverslip	nx1 matrix containing orientation values of each detected nucleus in an analyzed image set., defined as the angle of the

	major axis of the least-squares ellipse fitted to each nucleus . In units of degrees, with 360 degrees added to each orientation value.
excluded_files	Contains information regarding which images in a set were designated as unfit for analysis.

Summary_normal_defectives_ellipse.mat

avg_MNC_coverslip	Average mean negative curvature of detected nuclei in an image set.
avg_MNC_coverslip_adj	Average mean negative curvature of nuclei in an image set. Each value is normalized by the square root of the area of its corresponding nucleus.
avg_RC_coverslip	Average relative concavity of nuclei in an image set.
avg_Max_NC_adj_coverslip	Average maximum negative curvature of nuclei in an image set. Each value is normalized by the square root of the area of its corresponding nucleus.
MNC_fov_total	1xn matrix containing mean negative curvature values of each nucleus in an image set.
MNC_fov_total_adj	1xn matrix containing mean negative curvature values of each nucleus in an image set. Each value is normalized by the square root of the area of its corresponding nucleus.
normal_defective_percent	Percentage of abnormally shaped nuclei among all detected nuclei in an analyzed image set
RC_fov_total	1xn matrix containing relative concavity values of each nucleus in an image set.
Max_NC_fov_adj_total	1xn matrix containing maximum negative curvature values of each nucleus in an image set. Each value is normalized by the square root of the area of its corresponding nucleus.
normal_defective_count_total	1x2 matrix containing the number of nuclei classified as normal (column 1) and dysmorphic (column 2)
n_d_q_coordinates_total	nx3 matrix containing nuclear centroid x (column 1) and y (column 2) coordinates, and designation of each nucleus as normal or abnormal (column 3). ‘1’ indicates designation dysmorphic while ‘0’ indicates designation as normal.
abnormal_features_mean_median_std	6x3 matrix containing the mean (column 1), median (column

	2), and standard deviation (column 3) values for mean negative curvature (row 1), relative concavity (row 2), and max negative curvature (row 3), area in sq. microns (row 4), eccentricity (row 5), and perimeter in microns (row 6).
Areas_defectives_total	1xn matrix containing calculated areas (in sq. microns) of all nuclei in an image set classified by code as dysmorphic.
Eccentricities_defectives_total	1xn matrix containing calculated eccentricities of all nuclei in an image set classified by code as dysmorphic.
Perimeters_defectives_total	1xn matrix containing calculated perimeters (in microns) of all nuclei in an image set classified by code as dysmorphic.
Areas_normals_total	1xn matrix containing calculated areas (in sq. microns) of all nuclei in an image set classified by code as normal.
Eccentricities_normals_total	1xn matrix containing calculated eccentricities of all nuclei in an image set classified by code as normal
Perimeters_normals_total	1xn matrix containing calculated perimeters (in microns) of all nuclei in an image set classified by code as normal.
MNC_adj_normals_total	1xn matrix containing calculated mean negative curvature values of each nucleus in an image set classified as normal. Each value is normalized by the square root of the area of its corresponding nucleus.
RC_normals_total	1xn matrix containing calculated relative concavity values of each nucleus in an image set classified as normal.
Max_NC_adj_normals_total	1xn matrix containing maximum negative curvature values of each nucleus in an image set classified as normal. Each value is normalized by the square root of the area of its corresponding nucleus.
MNC_adj_defectives_total	1xn matrix containing mean negative curvature values of each nucleus in an image set classified as dysmorphic. Each value is normalized by the square root of the area of its corresponding nucleus.
RC_defectives_total	1xn matrix containing relative concavity values of each nucleus in an image set classified as dysmorphic.
Max_NC_adj_defectives_total	1xn matrix containing maximum negative curvature values of each nucleus in an image set classified as dysmorphic. Each value is normalized by the square root of the area of its corresponding nucleus.

NucleiDetect Code

```
%                            NUCLEIDETECT
%
% Arguments:
%     If analyzing new images:
%            .tiff image file FILENAME.tif of DAPI stained cardiac tissue
%
% Returns:
%            Figure of image with nuclei labeled and outlined and the
%            orientation displayed with a bar
%
%            .mat file named FILENAME_area_eccentricity_perimeter giving data
%            regarding nucleus in the image
%
%            .mat file named FILENAME_summary file containing averages for
parameters of nuclei
%            over an entire coverslip (all images selected at start)
%
%            J. Core  03/01/17

clear all
warning off images:initSize:adjustingMag
% Determine whether to save outputs
yesorno = input('Save Outputs? 0 = Yes, 1 = No \n');

% Conversion factor between image pixels and microns. Equals number of pixels
% in one micron
conversion_factor = 6.24;

pathfiles = 'E:\Jason\Final Testing\New folder9';
% Ask user to select files
[file,path]=uigetfile({'*.TIF';'*.*'},'Select Images...',
pathfiles,'MultiSelect','on');

% Set default threshold for all images
default_thresh = input('Enter default threshold for all images (0.4-1.1
recommended): ');

% Set case where input is multiple picture files, remember to cite later
NumFiles = length(file);

if iscell(file)
    NumFiles = length(file);
    for i=1:NumFiles
        temp = file{i};
        FileIndex{i} = temp;
    end
else
    NumFiles = 1;
    FileIndex{1} = file;
end

% Initialize matrix to contain parameters of nuclei of all images
yesorno2 = 1;
```

```
properlynamed = 1;
nonzero_orientation_angles_coverslip = [];
areas_total = [];
eccentricities_total = [];
perimeters_total = [];
excluded_files_count = 0;
excluded_files = [];

% Read image and store image name
for uu = 1:NumFiles
    customvalues = 0;
    [pathsr, name, ext] = fileparts(FileIndex{uu});
    filename = [path FileIndex{uu}];
    pathsr = path;
    A = imread(filename);

    % Check if images are colored
    if size(A,3) == 3
        A = rgb2gray(A);
    end

    % Initialize variables
    validcheck = 0;
    throwout = 0;
    exclude_numbers = 1000;
    double_nucleus_check = 0;
    threshscale = default_thresh;
    npoints = 100; % Set number of points to calculate curvature for
concavity detection. Controls detection of multiples, segmentation and number
of dents

    while validcheck < 1;
        close all
        clear MPC_fov_initial num_indents_initial MSE_fov_initial test2
mean_positive_curvature
        % Create variables in image detection
        npoints2 = 25; % Set number of points to fit circle to calculate
curvature. Controls MPC
        curve_threshold = .02;

        % Adjust the image intensity
        G=imadjust(A);
        G_size = size(G);

        % Convert image to black and white, fill in holes, and create matrix
of
        % X and Y values for each body.
        level = threshscale*graythresh(G);
        level2 = threshscale*graythresh(G);
        BW = im2bw(G,level);
        BW = imfill(BW,'holes');
        % Display original image
        %warning off images:initSize:adjustingMag
        imshow(G); hold on;
        %warning off images:initSize:adjustingMag
```

```
        % Begins new boundary extraction from image if previous data not
        % used
        [B,L] = bwboundaries(BW,'noholes');
        % Use regionprops to calculate orientation of bodies
        regions = regionprops(BW, 'Area', 'Perimeter');

        % Create regular vector to store area values so that they can be
        % removed more easily
        for counter = 1:length(regions)
            TempArea(counter) = regions(counter).Area;
            TempPerimeter(counter) = regions(counter).Perimeter;
        end
        TempArea = TempArea';
        TempPerimeter = TempPerimeter';

        % Convert areas of objects from pixels to micrometers
        TempArea = TempArea;
        TempPerimeter = TempPerimeter;

        % Remove any body with a perimeter of less than 8 microns
        micron_limit = 49.92/conversion_factor;
        remove_indices_2 = find(TempPerimeter<micron_limit);
        TempArea(remove_indices_2) = [];
        TempPerimeter(remove_indices_2)= [];
        B(remove_indices_2) = [];

        % Initialize variable used to keep track of number of outlines on
        % edge excluded
        increment = 0;
        % Initialize variable used to keep track of whether an outline
        % should be excluded
        Edgecheck = 0;

        % Remove bodies on edge of picture
        for i_counter = 1:(length(B)-increment)
            xy_points = cell2mat(B(i_counter-increment));
            x_points = xy_points(:,1);
            y_points = xy_points(:,2);
            if sum(y_points >= G_size(2))==0 && sum(y_points == 1)==0 &&
sum(x_points == 1)==0 && sum(x_points >= G_size(1))==0
            else
                % Store outlines designated as touching the edge for use
                % later in voronoi diagram
                Edgepoints(increment+1) = B(i_counter-increment);

                % Remove all other data regarding outlines on edge from
                % storage
                B(i_counter-increment) = [];
                TempArea(i_counter-increment)= [];
                TempPerimeter(i_counter-increment)= [];
                increment = increment+1;
                Edgecheck = 1;
            end
        end
```

```
        if Edgecheck == 0
            Edgepoints = [];
        end

        % Determine average area of the bodies in an image other
        % eliminations
        avg_area = mean(TempArea);

        % Remove bodies based on area
        remove_indices_3 = find(TempArea<avg_area/3);
        TempArea(remove_indices_3) = [];
        TempPerimeter(remove_indices_3)= [];
        B(remove_indices_3) = [];

        % Create variable to account for segmentation increasing number of
bodies
        valid_nucleus = [];

        % Get each valid nucleus outline in matrix B and calculate mean
curvature
        B = BoundaryRefine(A,B,level2);

        % Create struct variable to contain X and Y values of bodies
        struct_size = size(B,1);
        clear xy_values;
        xy_values(struct_size) = struct();
        clear ellipse_parameters;
        ellipse_parameters(struct_size) = struct();

        figure(1)
        for i = 1:length(B)
            % Convert X and Y cells into mat
            test = cell2mat(B(i,1));
            test2(i).xypairs = test;
            x_values = test(:,1);
            y_values = test(:,2);

            % Check for significant concavities

            % Evaluate curvature along the outline of the body
            outline = [x_values y_values];

            % Calculate curvature using polynomial fitting
            curve = curvature(outline(:,1),outline(:,2), 3, npoints,
npoints);
            curve_radius = curvature_circle(outline(:,1),outline(:,2), 3,
npoints2, npoints2);

            % Save values of outline curvature from poly fitting into new
            % variable
            curve_values(i).curve = curve;

            % Save values of outline curvature from circle fitting into new
```

```
            % variable
            curve_radius_values(i).curve = curve_radius;

            % Find all values of positive curvature in the outline
            % curvature from circle fitting
            curvature_radius_placehold = curve_radius;
            positive_curvature =
curvature_radius_placehold(find(curve_radius_values(i).curve>curve_threshold)
);

            % Calculate the mean of positive curvature values
            mean_positive_curvature(i) = mean(positive_curvature);
        end

        % Turn value of mean positive curvature from NaN to 0 for any
        % outline with no positive curvature to make valid
        mean_positive_curvature(find(isnan(mean_positive_curvature))) = 0;

        % Save MPC value for each nucleus into one variable
        MPC_fov_initial = mean_positive_curvature;

        % Calculate standard deviation of mean positive curvature across FOV
        %mean_positive_curvature(isnan(mean_positive_curvature)) = [];
        mean_mean_positive_curvature = mean(mean_positive_curvature);
        stddev = std(mean_positive_curvature);

        %Initialize struct array for outlines of multiple nuclei
        Multiplenuclei(1).xypairs = [0,0];
        multiple_indices = [];

        defect_numbers = [];
        for ii = 1:length(B)
            % Reset bleb check
            blebcheck = 0;
                % Clear indices so overlap does not occur
                % 'indices, indices2, and indices3 gives the indices of
points in the nucleus
                % outline where curvature spikes and becomes positive,
                % indicating a disruption in smoothness of outline
                clear indices indices2 indices3

                %get x and y points of each outline
                test = test2(ii).xypairs;
                x_values = test(:,1);
                y_values = test(:,2);

                % Identify points of positive and negative curvature

                % Get polynomial fit curvature values for an outline
                curve = curve_values(ii).curve;
                curve_index = curve > .003;
                indices = find(curve_index == 1);
                positive_curve = curve > curve_threshold;
                negative_curve = curve <= curve_threshold;
                positive_indices = find(positive_curve == 1);
```

```
                negative_indices = find(negative_curve == 1);

                % Get circle fit curvature values for an outline
                curve_radius = curve_radius_values(ii).curve;

                % Get indices for positive & negative circle fit curvature
values
                positive_curve_radius = curve_radius > curve_threshold;
                negative_curve_radius = curve_radius <= curve_threshold;
                positive_indices_radius = find(positive_curve_radius == 1);
                negative_indices_radius = find(negative_curve_radius == 1);

                % num_PC_points stores indices of points which are local
                % maxima of positive curvature. If there are none, it is 0
                if isempty(indices)
                    num_indents_initial(ii) = 0;
                else
                    % narrow down list of indices of points of positive
curvature to
                    % only those which are local maxima, so that one point
                    % corresponds to each disruption in curvature
                    indices2 = indices;
                    max1 = indices(1);
                    max_index = 1;
                    for increment  = 2:length(positive_indices)
                        % Scans along list of indices and compares points
                        % close together on the nucleus outline.
                        if positive_indices(increment)-
positive_indices(increment-1)<length(outline)/20
                            % Removes indices of point with smaller
                            % curvature value, until only the point with
                            % the maximum value in each peak remains
                            if curve(positive_indices(increment))<=max1
                                indices2(increment) = 0;
                           else
                               indices2(max_index) = 0;
                               max_index = increment;
                               max1 = indices(increment);
                            end
                        else
                            max1 = positive_indices(increment);
                        end
                    end

                    % Clear out the 0's corresponding to non-maximum
                    % points, only store the indices of maxima
                    inc = 0;
                    for increment2 = 1:length(indices2)
                        if indices2(increment2) ~= 0
                            inc = inc + 1;
                            indices3(inc) = indices2(increment2);
                        end

                    end
                    % Plot points of negative curvature over threshold
                    num_indents_initial(ii) = length(indices3);
```

```
                end

                % Clear indices to not interfere with finding indices of
                % next nucleus
                clear indices2 indices3

                % Find the points of maximum curvature
                curve_index = curve > .005;
                indices = find(curve_index == 1);

                % Check if object has been designated for removal and is
                % not already a double nucleus

                 if ~isempty(find(ii==exclude_numbers))
                    xy_values(ii).xypairs = test;
                    valid_nucleus(ii) = 0;
                    plot(y_values, x_values, 'r-', 'LineWidth', 2)
                    continue
                end

                % Record and plot nucleus if no significant concavities
                % found
                if isempty(indices)
                    xy_values(ii).xypairs = test;
                    valid_nucleus(ii) = 1;
                    plot(y_values, x_values, 'g-', 'LineWidth', 2)
                    continue
                end

                % narrow down list of indices of points of positive curvature
to
                % only those which are local maxima, so that one point
                % corresponds to each disruption in curvature
                indices2 = indices;
                max1 = indices(1);
                max_index = 1;
                for increment  = 2:length(indices)
                    % Scans along list of indices and compares points
                    % close together on the nucleus outline.
                    if indices(increment)-indices(increment-
1)<length(outline)/20
                        % Removes indices of point with smaller
                        % curvature value, until only the point with
                        % the maximum value in each peak remains
                        if curve(indices(increment))<=max1
                           indices2(increment) = 0;
                        else
                           indices2(max_index) = 0;
                           max_index = increment;
                           max1 = indices(increment);
                        end
                    else
                        max1 = indices(increment);
                    end
                end
```

```
            % Clear out the 0's corresponding to non-maximum
            % points, only store the indices of maxima
            inc = 0;
            for increment2 = 1:length(indices2)
                if indices2(increment2) ~= 0
                    inc = inc + 1;
                    % Indices3 contains the indices of points which are
                    % local maxima in positive curvature, each
                    % corresponding to a disruption in nucleus boundary
                    % curvature
                    indices3(inc) = indices2(increment2);
                end

            end

           % Requires more than 1 peak of positive curvature to recognize
           % as double nucleus
           if length(indices3) <= 1
               % Save outline of nucleus designated as single and valid
               xy_values(ii).xypairs = test;
               valid_nucleus(ii) = 1;
               % Plot values of positive and negative curvature as diff.
               % colors

               % Only plot boundaries as 1 color
               plot(y_values, x_values, 'g-', 'LineWidth', 2)
           else
               valid_nucleus(ii) = 0;
               xy_values(ii).xypairs = test;
               plot(y_values, x_values, 'r-', 'LineWidth', 2)
           end
        end


        % Access one set of x and y pairs from the struct array
        for i = 1:length(xy_values)
            test1 = xy_values(i).xypairs;
            x_values = test1(:,1);
            y_values = test1(:,2);

            % Area & perimeter calculated for each nucleus
            [geom, geom2, geom3] = polygeom(x_values,y_values);

            % Area & perimeter of each nucleus converted from pixels to um
(or um^2) before saving into
            % struct.
            areas(i) = geom(1)/conversion_factor^2;
            perimeters(i) = geom(4)/conversion_factor;

            % Fit ellipse to X and Y values, calculating parameters of the
            % ellipse
            values = 1:8;
            values = ellipsefit(x_values,y_values);
```

```
            % Store values related to fitted ellipse
            t = 0:.01:2*pi;
            x_1 = values(1);
            y_1 = values(2);
            values(3) = values(3)/conversion_factor;
            a1 = values(3);
            values(4) = values(4)/conversion_factor;
            b1 = values(4);
            values(6) = sqrt(1-(b1^2/a1^2));
            values(7) = areas(i);
            values(8) = perimeters(i);
            theta = values(5);
            if values(5) < 0
               values(5) = values(5) + pi;
            end

            % Create vectors corresponding to x and y values of points on
            % fitted ellipse
            x = x_1 + (a1*cos(t)*cos(theta)-
b1*sin(t)*sin(theta))*conversion_factor;
            y = y_1 +
(a1*cos(t)*sin(theta)+b1*sin(t)*cos(theta))*conversion_factor;
            xy_points = [x' y'];

            % Save parameters of each ellipse fitted to a body, as well as
            % its points
            ellipse_parameters(i).parameters = values;
            ellipse_parameters(i).outline = test1;
        end

        % Plot nuclei arrows
        for n=1:length(ellipse_parameters)
            % Set center of arrow as centroid of nucleus/fitted ellipse
            center = ellipse_parameters(n).parameters;
            h=text(center(2),center(1),num2str(n));
            set(h,'color','r');

            % Use calculated ellipse parameters to create best-fitting
            % arrow
            stats = ellipse_parameters(n).parameters;

            h=annotation('line');
            set(h,'parent',gca);
            angle = stats(5)*180/pi+90;
            if stats(3) > stats(4)
                majaxis = stats(3)*conversion_factor;
            else
                majaxis = stats(4*conversion_factor);
            end
            majaxis = majaxis*1.5;
            centroid = stats(1:2);

            if valid_nucleus(n) == 1
                % Math for setting length & orientation of arrow based on
```

```
                % ellipse parameters
                h=annotation('line');
                set(h,'parent',gca);
                set(h,'x',[(centroid(2)+majaxis*.8*cos(angle*pi/180))
(centroid(2)-majaxis*.8*cos(angle*pi/180))]);
                set(h,'y',[(centroid(1)-(majaxis*.8*sin(angle*pi/180)))
(centroid(1)+(majaxis*.8*sin(angle*pi/180)))]);
                set(h,'Color','r');
            end
        end

        hold off;

        % Show raw nuclei image with nothing plotted
        figure(2)
        imshow(G)
        % Show nuclei image converted to binary based on threshold
        figure(3)
        imshow(BW)

        % Prompt user to re-analyze with different values or accept image
        figure(1)
        w = input('Accept Values? (yes = 0, no: input new threshold =  1,
input new overlap detection constant = 2 throw out objects = 6,\n throw out
image = 13, reset = 22): ');
        if w == 0
            disp('Image Values accepted' )
            validcheck = 1;
        % Option for throwing out image and designating its data as invalid,
but still saving
        elseif w == 13
            validcheck = 1;
            throwout = 1;
            excluded_files_count = excluded_files_count + 1;
            excluded_files(excluded_files_count).name = name;
        % Option for designating specific nuclei to manually exclude from
        % analysis
        elseif w == 6
            exclude_numbers = str2num(input('Enter object numbers to exclude:
', 's'));
            final_exclusion_indices = double_nucleus_check;
            defect_numbers = [];
            disp('Re-analyze imaging...')
        % Option for resetting all conditions and re-analyzing image with
default
        % settings
        elseif w == 22
            threshscale = default_thresh;
            npoints = 40;
            customvalues = 0;
            clear multiple_indices
            defect_numbers = [];
            exclude_numbers = [];
        % Option for re-analyzing under different threshold and
        % segmentation constant
        elseif w == 2
```

```
            disp('Re-analyze imaging...')
            customvalues = 1;
            clear MPC_fov_initial MSE_fov_initial num_indents_initial
            disp(['Values set: Initial threshold = ' num2str(threshscale) '
and Overlap Detection Value = '  num2str(npoints)])
            npoints = input('Enter Overlap Detection Value (70-200
recommended, more points = less automatic exclusion) ');
            if npoints < 8
                disp('Enter a number greater than or equal to 8')
                npoints = input('Enter Overlap Detection Value (70-200
recommended, higher value = less detection) ');
            end
        else
            disp('Re-analyze imaging...')
            customvalues = 1;
            clear MPC_fov_initial MSE_fov_initial num_indents_initial
            disp(['Values set: Initial threshold = ' num2str(threshscale) '
and Overlap Detection Value = '  num2str(npoints)])
            threshscale = input('Enter Threshold (0.5-1 recommended): ');
            if threshscale > 2
                disp('Enter lower number./n')
                threshscale = input('Enter Threshold (0.5-1 recommended): ');
            end
        end
    end

    % Initialize matrices for valid nuclei data
    xy_values_final = [];
    ellipse_parameters_final = [];
    clear areas perimeters

    % Separate valid nuclei data from excluded nuclei data
    counts2 = 0;
    counts3 = 0;
    for counts = 1:length(valid_nucleus)
        if valid_nucleus(counts) == 1
            counts2 = counts2 + 1;
            xy_values_final(counts2).xypairs = xy_values(counts).xypairs;
            ellipse_parameters_final(counts2).parameters =
ellipse_parameters(counts).parameters;
            B_new(counts2) = B(counts);
        else
            counts3 = counts3 + 1;
            xy_values_excluded(counts3).xypairs = xy_values(counts).xypairs;
            ellipse_parameters_excluded(counts3).parameters =
ellipse_parameters(counts).parameters;
            B_excluded(counts3) = B(counts);
        end
    end

    % Creating orientation matrix and Voronoi diagram
    orientim_perp(1:size(G,1),1:size(G,2)) = 0;
    for m=1:length(ellipse_parameters_final)
        stats = ellipse_parameters_final(m).parameters;
        angle = stats(5);
        if angle < 0
```

```
            angle = angle + pi;
        end
        % Place orientation angle values of each nucleus into
        % the orientim_perp matrix, which has the same dimensions as
        % the image
        orientim_perp(round(stats(1)),round(stats(2))) = angle+2*pi;

        % Store coordinates of centroid of each nucleus
        centroids(m,1) = stats(1);
        centroids(m,2) = stats(2);
    end

    % Store the orientim_perp as voronoi_orientim_perp for saving in
    % voronoi input file
    voronoi_orientim_perp = orientim_perp;
    regionstats(1).Orientation_Matrix = orientim_perp;

    % Store original image in struct
    regionstats(1).Image = G;

    % Temporary method for combining ellipse_parameters and regionstats in
order
    % to create one struct to return
    for n=1:length(ellipse_parameters_final)
        values = ellipse_parameters_final(n).parameters;
        regionstats(n).Centroid = [values(1) values(2)];
        if values(5) < 0
            values(5) = values(5) + pi;
        end
        regionstats(n).Orientation = values(5);
        if values(3)>values(4)
            regionstats(n).MajorAxisLength = values(3);
        else
            regionstats(n).MajorAxisLength = values(4);
        end
    end

    % Store area and eccentricity of each nucleus
    for ty = 1:length(ellipse_parameters_final)
        params = ellipse_parameters_final(ty).parameters;
        areas(ty) = params(7);
        perimeters(ty) = params(8);
        eccentricities(ty) = params(6);
    end

    % Eliminate dimensions of areas and perimeters being randomly flipped
    if size(areas,1) ~= 1
        areas = areas';
    end
    if size(perimeters,1) ~= 1
        perimeters = perimeters';
    end

    % Create matrices containing average parameters and all parameters of
    % each nucleus
```

```
    if throwout == 0
        areas_total = [areas_total areas];
        perimeters_total = [perimeters_total perimeters];
        eccentricities_total = [eccentricities_total eccentricities];
    end

    % Calculate average parameters across a FOV
    avg_areas_fov(uu) = mean(areas);
    avg_eccentricities_fov(uu) = mean(eccentricities);
    avg_perimeters_fov(uu) = mean(perimeters);

    % Keep nonzero values of matrix of orientation angles
    orientation_fov = orientim_perp(:);
    nonzero_orientation_fov = orientation_fov(find(orientation_fov));
    nonzero_orientation_angles_fov = rad2deg(nonzero_orientation_fov);
    if throwout == 0
        nonzero_orientation_angles_coverslip =
[nonzero_orientation_angles_coverslip; nonzero_orientation_angles_fov ];
    end
    orientation_voronoi_fov = voronoi_orientim_perp(:);

     % Convert radians to degrees
   nonzero_orientation_angles_fov = rad2deg(nonzero_orientation_fov);

    % Create new names for final image and .mat file
    string1 = [path name '_final_image'];
    string3 = [path name '_area_eccentricity_perimeter.mat'];
    string4 = [path 'summary'];
    string5 = [path name '_voronoi_input'];

    if yesorno == 0 && throwout == 0
        % Save FILENAME.mat file
        % Adjust Figure to Eliminate White Border in Saved Image
        figure(1)
        set(gca, 'position', [0 0 1 1], 'units', 'normalized')
        set(gcf, 'PaperUnits', 'inches', 'PaperPosition', [0 0 13.44 10.24]);
        print('-dtiff', string1, '-r100');
        %save (string5, 'orientim_perp', 'valid_nucleus', 'centroids',
'Edgepoints', 'ellipse_parameters');
        save (string3, 'B_new', 'areas', 'eccentricities', 'perimeters',
'nonzero_orientation_angles_fov', 'level2', 'throwout', 'conversion_factor');
    end

    % Clear all variables except necessary ones for the current image, move
to next
    clearvars -except conversion_factor all_parameters excluded_files_count
default_thresh image_colored excluded_files Edgepoints2 curve_values p
voronoi_orientim_perp orientation_voronoi_fov properlynamed yesorno yesorno2
file file2 path path2 Numfiles Numfiles2 FileIndex FileIndex2 yesorno
avg_areas_fov avg_eccentricities_fov avg_perimeters_fov string4
nonzero_orientation_angles_coverslip areas_total eccentricities_total
perimeters_total imagedate stringg plate imagename curve_values

end
```

```
% Calculate average parameter values of nuclei across an entire coverslip
% at the end of a batch of images
avg_areas_coverslip = mean(areas_total);
avg_eccentricities_coverslip = mean(eccentricities_total);
avg_perimeters_coverslip = mean(perimeters_total);

% Save Summary File for Data Concerning Averages over all FOVs
if yesorno == 0
save (string4, 'avg_areas_coverslip', 'avg_eccentricities_coverslip',
'avg_perimeters_coverslip', 'nonzero_orientation_angles_coverslip',
'areas_total', 'eccentricities_total', 'perimeters_total', 'excluded_files')
end
%clear all
%close all
warning on images:initSize:adjustingMag
msgbox('Operation Completed')
```

NucleiClassify Code

```
%                           Nuclei Defect
%
% Uses outlines (2D sets of points generated by NucleiDetect) of nuclei to
% determine whether the nuclei have normal morphology, or whether they
% possess irregularities which designate them as questionable or defective.
%
% Arguments:
%      .mat file FILENAME_area_eccentricity_perimeter.mat
%
%      .tif image corresponding to the
%      FILENAME_area_eccentricity_perimeter.mat file
%
% Returns:
%      updated .mat file named FILENAME_area_eccentricity_perimeter with
%      original data plus calculated shape parameter values
%
%      .tif image containing nuclei colored based on classification
%
%      .mat summary file containing average MNC, relative concavity,
%      and Max NC values across all nuclei in the coverslip, proportion of
%      defective nuclei, and their coordinates in their respective images.
%
%            J. Core  04/01/2017

close all
clear all

pathfiles = 'E:\Jason\Final Testing\New folder9';

if ~exist('conversion_factor')
    conversion_factor = 6.24;
end

% Determine whether to save nuclei with colored outlines or colored
% interiors
```

```
color_options = input('Create .tif nuclei images with colored outlines (0) or
colored interiors (1)?');

% Select .mat files for analysis
[file,path]=uigetfile({'*.mat';'*.*'},'Select area_eccentricity_perimeter
files...', pathfiles,'MultiSelect','on');

% Set case where input is multiple picture files
NumFiles = length(file);
if iscell(file)
    for i=1:NumFiles
        temp = file{i};
        FileIndex{i} = temp;
    end
else
    NumFiles = 1;
    FileIndex{1} = file;
end

% Select .tif files for analysis
[file2,path2]=uigetfile({'*.TIF';'*.*'},'Select .tif files...',
pathfiles,'MultiSelect','on');
% Set case where input is multiple picture files
NumFiles2 = length(file2);
if iscell(file2)
    for i=1:NumFiles2
        temp2 = file2{i};
        FileIndex2{i} = temp2;
    end
else
    NumFiles2 = 1;
    FileIndex2{1} = file2;
end

% Initialize matrix to contain parameters of nuclei of all images
n_d_q_coordinates_total = [];
MNC_fov_total = [];
MNC_fov_adj_total = [];
RC_fov_total = [];
Max_NC_fov_adj_total = [];
Areas_defectives_total = [];
Eccentricities_defectives_total = [];
Perimeters_defectives_total = [];
MNC_adj_defectives_total = [];
RC_defectives_total = [];
Max_NC_adj_defectives_total = [];
Areas_normals_total = [];
Eccentricities_normals_total = [];
Perimeters_normals_total = [];
MNC_adj_normals_total = [];
RC_normals_total = [];
Max_NC_adj_normals_total = [];
Hull_area_diff_total = [];
num_indents_total = [];
normal_defective_count_total = [0 0];
```

```
% Read image and store image name
for uu = 1:NumFiles
    customvalues = 0;
    % Load .mat file
    [pathsr, name, ext] = fileparts(FileIndex{uu});
    filename = [path FileIndex{uu}];
    pathsr = path;
    load(filename);

    % Load .tif image corresponding to .mat file
    [pathsr2, name2, ext2] = fileparts(FileIndex2{uu});
    filename2 = [path2 FileIndex2{uu}];
    A = imread(filename2);

    % Check if images are colored
    if size(A,3) == 3
        A = rgb2gray(A);
    end

    A_original = A;
    A = imadjust(A);
    imshow(A)
    figure(2)
    imshow(A)
    figure(1)

    % Skip to next image and mat file if data was designated as bad
    if throwout == 1;
        excluded_files_count = excluded_files_count + 1;
        excluded_files(excluded_files_count).name = name;
        continue
    end

    % Clear matrices from previous file
    clear MPC_fov PC_fov RC_fov max_NC num_indents normal_defective_count ...
        normals defectives questionables test2 mean_positive_curvature
sum_positive_curvature curve_values curve_radius_values

    % Initialize variables
    n_d_q_coordinates = [];
    Options.nPoints = 1000; % Set number of points that final nucleus outline
will contain.
    npoints2 = 25; % Set number of points to fit polynomial to calculate
curvature. Controls MPC
    throwout = 0;
    curve_threshold = 0;
    hull_threshold = 0;
    npoints = 40; % Set number of points to calculate curvature for concavity
detection. Controls detection of multiples, segmentation and number of dents

    B_new = BoundaryRefine(A_original,B_new,level2);
    for i = 1:length(B_new)
        clear indices indices2 indices3
        % Convert X and Y cells into mat
        test = cell2mat(B_new(i));
```

```
        test2(i).xypairs = test;
        x_values = test(:,1);
        y_values = test(:,2);

        % Evaluate curvature along the outline of the body
        outline = [x_values y_values];

        % Calculate curvature using polynomial fitting
        curve = curvature(outline(:,1),outline(:,2), 3, npoints, npoints);
        curve_radius = curvature_circle(outline(:,1),outline(:,2), 3,
npoints2, npoints2);

        % Find point of maximum negative curvature. If only positive
        % curvature exists, set to 0
        if max(curve_radius) < 0
            max_NC(i) = 0;
        else
            max_NC(i) = max(curve_radius);
        end

        % Save values of outline curvature from poly fitting into new
        % variable
        curve_values(i).curve = curve;

        % Save values of outline curvature from circle fitting into new
        % variable
        curve_radius_values(i).curve = curve_radius;

        % Find all values of positive curvature in the outline
        % curvature from circle fitting
        curvature_radius_placehold = curve_radius;
        positive_curvature =
curvature_radius_placehold(find(curve_radius_values(i).curve>curve_threshold)
);

        % Calculate the mean of positive curvature values
        mean_positive_curvature(i) = mean(positive_curvature);
        sum_positive_curvature(i) = sum(positive_curvature);

        % Begin finding numbers of indents
        curve_index = curve > curve_threshold;
        indices = find(curve_index == 1);
        positive_curve = curve > curve_threshold;
        negative_curve = curve <= curve_threshold;
        positive_indices = find(positive_curve == 1);
        negative_indices = find(negative_curve == 1);
        % num_PC_points stores indices of points which are local
        % maxima of positive curvature. If there are none, it is 0
        if isempty(indices)
            num_indents(i) = 0;
        else
            % narrow down list of indices of points of positive curvature to
            % only those which are local maxima, so that one point
            % corresponds to each disruption in curvature
```

```
            indices2 = indices;
            max1 = indices(1);
            max_index = 1;
            for increment  = 2:length(positive_indices)
                % Scans along list of indices and compares points
                % close together on the nucleus outline.
                if positive_indices(increment)-positive_indices(increment-
1)<length(outline)/20
                    % Removes indices of point with smaller
                    % curvature value, until only the point with
                    % the maximum value in each peak remains
                    if curve(positive_indices(increment))<=max1
                        indices2(increment) = 0;
                    else
                        indices2(max_index) = 0;
                        max_index = increment;
                        max1 = indices(increment);
                    end
                else
                    max1 = positive_indices(increment);
                end
             end

             % Clear out the 0's corresponding to non-maximum
             % points, only store the indices of maxima
             inc = 0;
             for increment2 = 1:length(indices2)
                 if indices2(increment2) ~= 0
                     inc = inc + 1;
                     indices3(inc) = indices2(increment2);
                 end
             end
             num_indents(i) = length(indices3);
         end
    end

    % Turn value of mean positive curvature from NaN to 0 for any
    % outline with no positive curvature to make valid
    mean_positive_curvature(find(isnan(mean_positive_curvature))) = 0;
    sum_positive_curvature(find(isnan(sum_positive_curvature))) = 0;
    % Save MPC value for each nucleus into one variable
    MNC_fov = mean_positive_curvature;
    PC_fov = sum_positive_curvature;

    % Access one set of x and y pairs from the struct array
    for i = 1:length(test2)
        test1 = test2(i).xypairs;
        x_values = test1(:,1);
        y_values = test1(:,2);

        % Fit ellipse to X and Y values, calculating parameters of the
        % ellipse
        values = 1:8;
        values = ellipsefit(x_values,y_values);

        % Store values related to fitted ellipse
```

```
        t = 0:.01:2*pi;
        x_1 = values(1);
        y_1 = values(2);
        values(3) = values(3)/conversion_factor;
        a1 = values(3);
        values(4) = values(4)/conversion_factor;
        b1 = values(4);
        values(6) = sqrt(1-(b1^2/a1^2));
        values(7) = areas(i);
        values(8) = perimeters(i);
        theta = values(5);
        if values(5) < 0
           values(5) = values(5) + pi;
        end

        % Store centroids of each nucleus in matrix. Will later add a third
        % column designating each centroid as normal, defective or
        % questionable

        n_d_q_coordinates(i,1:2) = [x_1 y_1];

        % Create vectors corresponding to x and y values of points on
        % fitted ellipse
        x = x_1 + (a1*cos(t)*cos(theta)-
b1*sin(t)*sin(theta))*conversion_factor;
        y = y_1 +
(a1*cos(t)*sin(theta)+b1*sin(t)*cos(theta))*conversion_factor;
        xy_points = [x' y'];

        % Save parameters of each ellipse fitted to a body, as well as
        % its points
        ellipse_parameters(i).parameters = values;
        ellipse_parameters(i).outline = xy_points;

        % Compute difference in area between nucleus area and area of its
        % convex hull
        nucleus_convex_hull = convhull(x_values,y_values);
        hull_values = [x_values(nucleus_convex_hull)
y_values(nucleus_convex_hull)];
        hull_area = polyarea(hull_values(:,1), hull_values(:,2));
        nucleus_area = polyarea(x_values,y_values);
        RC_fov(i) =  (hull_area - nucleus_area)/hull_area;
    end


    % Sort nuclei into normal, questionable or defective groups based
    % on MPC & MSE
    normals = [];
    questionables_d = [];
    questionables_n = [];
    defectives = [];
    MNC_fov_adj = MNC_fov.*sqrt(areas*conversion_factor^2);
    Max_NC_fov_adj = max_NC.*sqrt(areas*conversion_factor^2);
    MPC_MSE_pairs = [MNC_fov_adj' RC_fov' Max_NC_fov_adj'];
```

```
    % Set Limits for Abnormality Check
    box_lower_MNC = .67;
    box_upper_MNC = 1.5634;
    box_lower_RC = .0025;
    box_upper_RC = .0201;
    box_lower_MaxNC = 1.3109;
    box_upper_MaxNC = 3.486;
    box_inner_limit_MNC = 1.3692;
    box_inner_limit_RC = .0048;
    box_inner_limit_MaxNC = 2.6703;


    % Use shape features to determine abnormal or normal shape
    for tt = 1:size(MPC_MSE_pairs,1)
        if MPC_MSE_pairs(tt,1) < box_lower_MNC || MPC_MSE_pairs(tt,2) <
box_lower_RC || MPC_MSE_pairs(tt,3) < box_lower_MaxNC
            normals(end+1) = tt;
        elseif MPC_MSE_pairs(tt,1) >= box_lower_MNC && MPC_MSE_pairs(tt,1) <=
box_upper_MNC && MPC_MSE_pairs(tt,2) >= box_lower_RC && MPC_MSE_pairs(tt,2)
<= box_upper_RC && MPC_MSE_pairs(tt,3) >= box_lower_MaxNC &&
MPC_MSE_pairs(tt,3) <= box_upper_MaxNC
            if MPC_MSE_pairs(tt,1) >= box_inner_limit_MNC &&
MPC_MSE_pairs(tt,2) >= box_inner_limit_RC || MPC_MSE_pairs(tt,1) >=
box_inner_limit_MNC && MPC_MSE_pairs(tt,3) >= box_inner_limit_MaxNC ||
MPC_MSE_pairs(tt,2) >= box_inner_limit_RC && MPC_MSE_pairs(tt,3) >=
box_inner_limit_MaxNC
                questionables_d(end+1) = tt;
            else
                questionables_n(end+1) = tt;
            end
        elseif MPC_MSE_pairs(tt,1) > box_upper_MNC || MPC_MSE_pairs(tt,2) >
box_upper_RC || MPC_MSE_pairs(tt,3) > box_upper_MaxNC
            defectives(end+1) = tt;
        else
            disp('exception')
        end
    end

    % Create matrix containing raw number of nuclei designated as normal
    % and abnormal
    normal_defective_count = [length(normals)+length(questionables_n)
length(questionables_d)+length(defectives)];

    % Tag centroids of nuclei as either normal or defective
    n_d_q_coordinates(normals,3) = 0;
    n_d_q_coordinates(questionables_n,3) = 0;
    n_d_q_coordinates(questionables_d,3) = 1;
    n_d_q_coordinates(defectives,3) = 1;

    if color_options == 1
        ndq = n_d_q_coordinates(:,3);
        colorimage = ColoredNuclei(A_original,B_new,level2,ndq);
    end

    % Plot the detected outlines of each nucleus and tag it with its
    % normal, questionable, or defective status
```

```
    hold on
    for ii = 1:length(B_new)
        test = cell2mat(B_new(ii));
        x_values = test(:,1);
        y_values = test(:,2);
        MPC_text = num2str(MPC_MSE_pairs(ii,1));
        Max_text = num2str(MPC_MSE_pairs(ii,3));
        Hull_text = num2str(MPC_MSE_pairs(ii,2));
        fontsizer = 10;

        % Outline nuclei with red (abnormal) or green (normal)
        if n_d_q_coordinates(ii,3) == 0
            plot(y_values, x_values, 'g', 'LineWidth', 3)
            centroid_x = n_d_q_coordinates(ii,1)+55;
            centroid_y = n_d_q_coordinates(ii,2)-35;
        elseif n_d_q_coordinates(ii,3) == 1
            plot(y_values, x_values, 'r', 'LineWidth', 3)
            centroid_x = n_d_q_coordinates(ii,1)+55;
            centroid_y = n_d_q_coordinates(ii,2)-35;
        end
    end

    % Get area, eccentricity, perimeter, and feature values of all nuclei
    % in image designated as abnormal
    defective_indices = find(n_d_q_coordinates(:,3)==1);
    Areas_defectives = areas(defective_indices)';
    Eccentricities_defectives = eccentricities(defective_indices)';
    Perimeters_defectives = perimeters(defective_indices)';
    MNC_adj_defectives = MPC_MSE_pairs([defectives questionables_d], 1);
    RC_defectives = MPC_MSE_pairs([defectives questionables_d], 2);
    Max_NC_adj_defectives = MPC_MSE_pairs([defectives questionables_d], 3);

    % Get area, eccentricity, perimeter, and feature values of all nuclei
    % in image designated as normal
    normal_indices = find(n_d_q_coordinates(:,3)==0);
    Areas_normals = areas(normal_indices)';
    Eccentricities_normals = eccentricities(normal_indices)';
    Perimeters_normals = perimeters(normal_indices)';
    MNC_adj_normals = MPC_MSE_pairs([normals questionables_n], 1);
    RC_normals = MPC_MSE_pairs([normals questionables_n], 2);
    Max_NC_adj_normals = MPC_MSE_pairs([normals questionables_n], 3);

    % Add these feature values to vector containing information over all
    % images/the coverslip
    if throwout == 0
        n_d_q_coordinates_total = [n_d_q_coordinates_total;
n_d_q_coordinates];
        Areas_defectives_total = [Areas_defectives_total Areas_defectives'];
        Eccentricities_defectives_total = [Eccentricities_defectives_total
Eccentricities_defectives'];
        Perimeters_defectives_total = [Perimeters_defectives_total
Perimeters_defectives'];
        MNC_adj_defectives_total = [MNC_adj_defectives_total;
MNC_adj_defectives];
        RC_defectives_total = [RC_defectives_total; RC_defectives];
```

```
        Max_NC_adj_defectives_total = [Max_NC_adj_defectives_total;
Max_NC_adj_defectives];

        Areas_normals_total = [Areas_normals_total Areas_normals'];
        Eccentricities_normals_total = [Eccentricities_normals_total
Eccentricities_normals'];
        Perimeters_normals_total = [Perimeters_normals_total
Perimeters_normals'];
        MNC_adj_normals_total = [MNC_adj_normals_total; MNC_adj_normals];
        RC_normals_total = [RC_normals_total; RC_normals];
        Max_NC_adj_normals_total = [Max_NC_adj_normals_total;
Max_NC_adj_normals];

        MNC_fov_adj_total = [MNC_fov_adj_total MNC_fov_adj];
        MNC_fov_total = [MNC_fov_total MNC_fov];
        RC_fov_total = [RC_fov_total RC_fov];
        Max_NC_fov_adj_total = [Max_NC_fov_adj_total Max_NC_fov_adj];
        num_indents_total = [num_indents_total num_indents];
        normal_defective_count_total =
[normal_defective_count_total(1)+normal_defective_count(1)...
            normal_defective_count_total(2)+normal_defective_count(2)];
    end

    % Create file names of output
    string1 = [path name '_auto_count'];
    string3 = [path name];

    % Adjust output image size and save
    if color_options == 1
        figure(1)
        imshow(colorimage)
    end
    set(gca, 'position', [0 0 1 1], 'units', 'normalized')
        set(gcf, 'PaperUnits', 'inches', 'PaperPosition', [0 0 13.44 10.24]);
        print('-dtiff', string1, '-r100');

    % Re-save area_eccentricity_perimeter.mat file containing new data
    % related to defective nuclei (MNC, MaxNC, relative concavity)
    save (string3, 'B_new', 'level2', 'n_d_q_coordinates', 'areas',
'eccentricities', ...
        'perimeters', 'MNC_fov', 'MNC_fov_adj', 'normal_defective_count',...
        'nonzero_orientation_angles_fov', 'throwout');

end

% Average, median, and standard deviation values over all abnormal nuclei in
all selected images/the coverslip
avg_Areas_defectives_coverslip = mean(Areas_defectives_total);
avg_Eccentricities_defectives_coverslip =
mean(Eccentricities_defectives_total);
avg_Perimeters_defectives_coverslip = mean(Perimeters_defectives_total);
avg_MNC_adj_defectives_coverslip = mean(MNC_adj_defectives_total);
avg_RC_defectives_coverslip = mean(RC_defectives_total);
avg_Max_NC_adj_defectives_coverslip = mean(Max_NC_adj_defectives_total);
median_MNC_defectives_coverslip = median(MNC_adj_defectives_total);
```

```
median_RC_defectives_coverslip = median(RC_defectives_total);
median_Max_NC_defectives_coverslip = median(Max_NC_adj_defectives_total);
median_Areas_defectives_coverslip = median(Areas_defectives_total);
median_Eccentricities_defectives_coverslip =
median(Eccentricities_defectives_total);
median_Perimeters_defectives_coverslip = median(Perimeters_defectives_total);
std_MNC_defectives_coverslip = std(MNC_adj_defectives_total);
std_RC_defectives_coverslip = std(RC_defectives_total);
std_Max_NC_defectives_coverslip = std(Max_NC_adj_defectives_total);
std_Areas_defectives_coverslip = std(Areas_defectives_total);
std_Eccentricities_defectives_coverslip =
std(Eccentricities_defectives_total);
std_Perimeters_defectives_coverslip = std(Perimeters_defectives_total);

% Put values for abnormal nuclei in matrix form
abnormal_features_mean_median_std = [avg_MNC_adj_defectives_coverslip
median_MNC_defectives_coverslip std_MNC_defectives_coverslip;...
    avg_RC_defectives_coverslip median_RC_defectives_coverslip
std_RC_defectives_coverslip;...
    avg_Max_NC_adj_defectives_coverslip median_Max_NC_defectives_coverslip
std_Max_NC_defectives_coverslip;...
    avg_Areas_defectives_coverslip median_Areas_defectives_coverslip
std_Areas_defectives_coverslip;...
    avg_Eccentricities_defectives_coverslip
median_Eccentricities_defectives_coverslip
std_Eccentricities_defectives_coverslip;...
    avg_Perimeters_defectives_coverslip
median_Perimeters_defectives_coverslip std_Perimeters_defectives_coverslip];



% Average shape feature values over all nuclei in selected images/the
coverslip
avg_MNC_coverslip = mean(MNC_fov_total);
avg_MNC_coverslip_adj = mean(MNC_fov_adj_total);
avg_RC_coverslip = mean(RC_fov_total);
avg_Max_NC_adj_coverslip = mean(Max_NC_fov_adj_total);

% Find percentage of nuclei designated as abnormal in all images/the
coverslip
normal_defective_percent =
(normal_defective_count_total(2)/(sum(normal_defective_count_total)));

% Save summary file containing data over all images/the coverslip
string5 = [path 'summary_normal_defectives'];
save(string5, 'MNC_fov_total', 'MNC_fov_adj_total',
'n_d_q_coordinates_total', ...
    'avg_MNC_coverslip', 'avg_MNC_coverslip_adj',
'normal_defective_count_total', ...
    'normal_defective_percent', 'abnormal_features_mean_median_std',
'Areas_defectives_total', 'Eccentricities_defectives_total', ...
    'Perimeters_defectives_total', 'RC_fov_total', 'Max_NC_fov_adj_total',
'avg_RC_coverslip', 'avg_Max_NC_adj_coverslip', ...
    'Areas_normals_total', 'Eccentricities_normals_total',
'Perimeters_normals_total', ...
    'MNC_adj_normals_total', 'RC_normals_total', 'Max_NC_adj_normals_total',
...
```

```
    'MNC_adj_defectives_total', 'RC_defectives_total',
'Max_NC_adj_defectives_total')
msgbox('Operation Completed')
```
