# Supplementary material for: Age of heart disease presentation and dysmorphic nuclei in patients with LMNA mutations
Source: PLoS One. 2017 Nov 17;12(11):e0188256. doi: 10.1371/journal.pone.0188256 (PMC5693421; doi:10.1371/journal.pone.0188256)

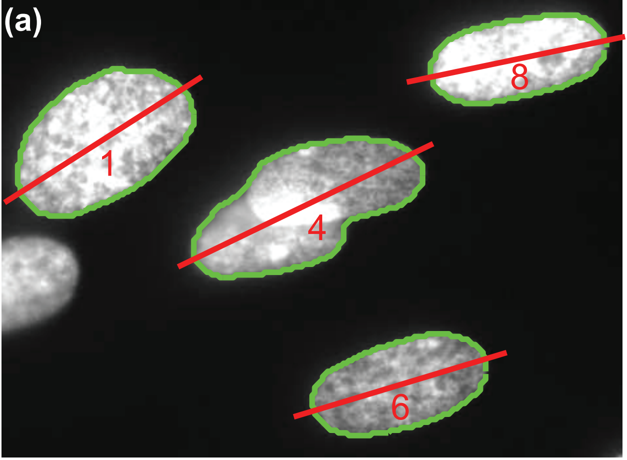

(b)

```
Accept Values?
yes = 0,
no: input new threshold =  1,
input new overlap detection constant = 2
throw out objects = 6,
throw out image = 13,
reset = 22: 6
Enter object numbers to exclude: 4
Re-analyze imaging...
```

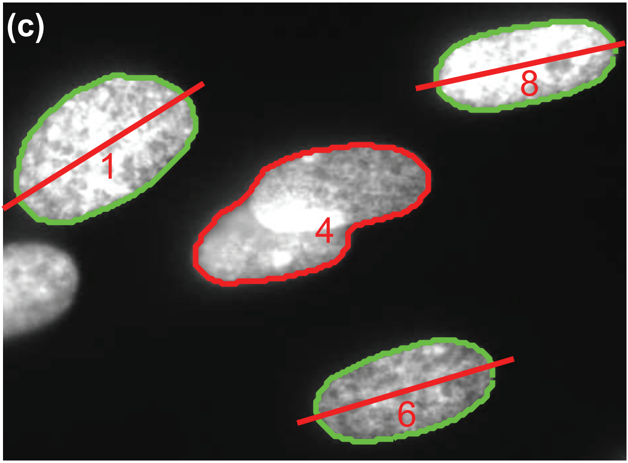

Supplement: S2 Fig — a Following automatic segmentation of nuclei, an image is displayed with all detected nuclei highlighted and numbered, including one undetected pair of overlapping nuclei; b User is prompted to choose an action after reviewing the displayed image, entering ‘6’ in order to exclude the overlapping nuclei from further analysis, and then entering the number corresponding to that body in the image; c The overlapping nuclei are excluded from analysis, and highlighted in red. (PDF) [file pone.0188256.s010.pdf]
