# Supplementary figures and images for: Age of heart disease presentation and dysmorphic nuclei in patients with LMNA mutations
Source: PLoS One. 2017 Nov 17;12(11):e0188256. doi: 10.1371/journal.pone.0188256 (PMC5693421; doi:10.1371/journal.pone.0188256)

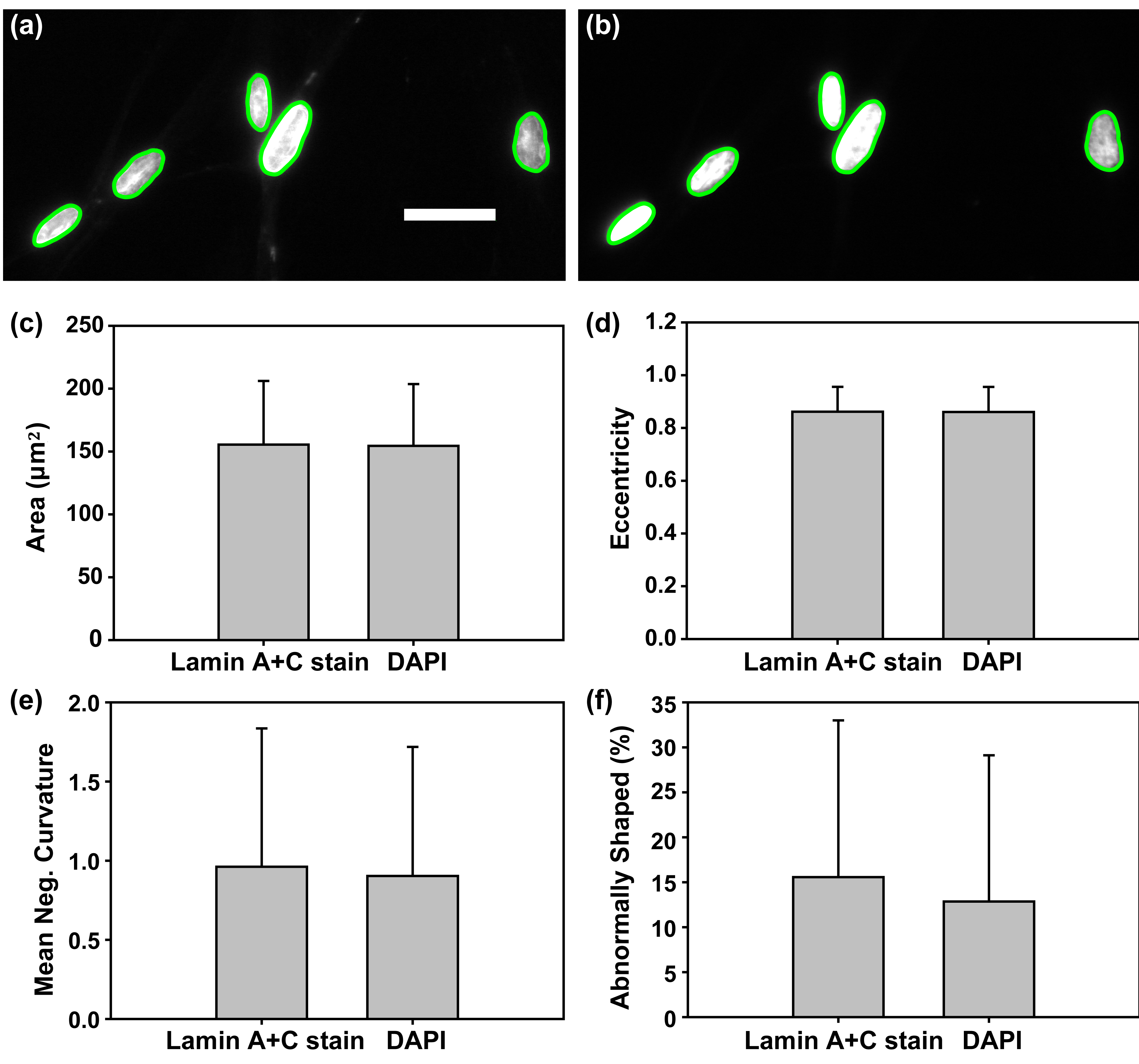

Supplement: S3 Fig — Nuclei were visualized with both a Lamin A+C immunofluorescence label and b DAPI, and then segmented by the image analysis Matlab code; c Average area, d average eccentricity, and e average mean negative curvature were then calculated based on nuclear boundaries in both the DAPI and Lamin A+C stain images (n = 48 nuclei). f The percentage of nuclei designated as dysmorphic was also calculated for all images and averaged by stain type (n = 12 images). Error bars represent one standard deviation. Scale bar: 25 μm. (TIF) [file pone.0188256.s011.tif]

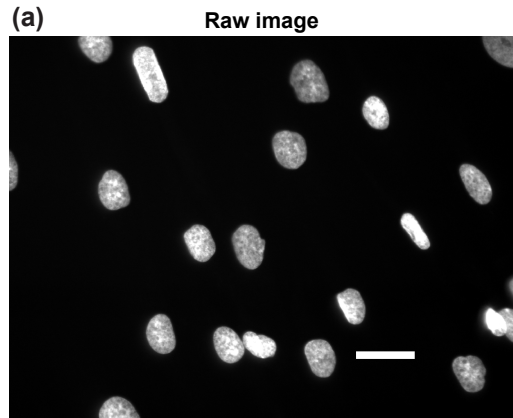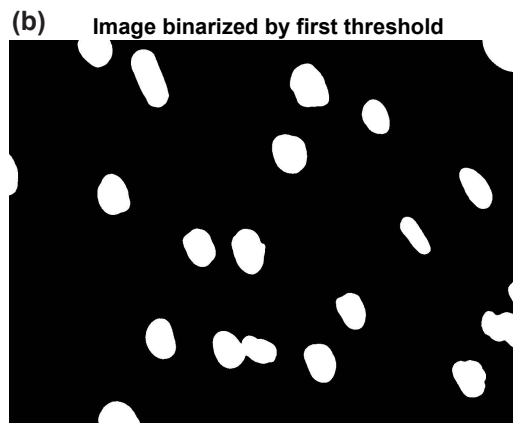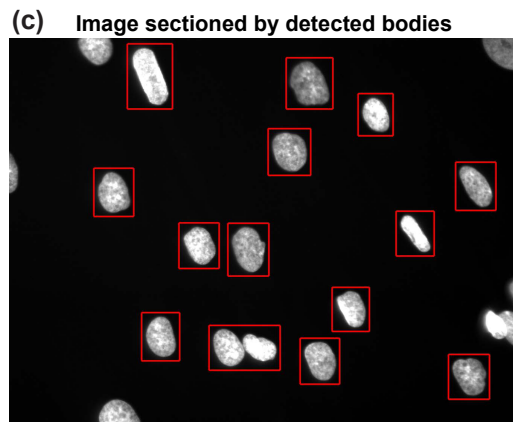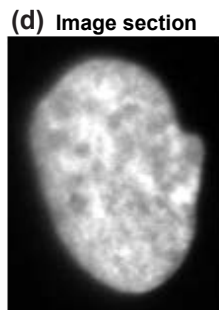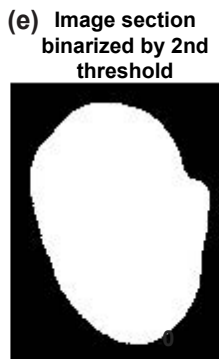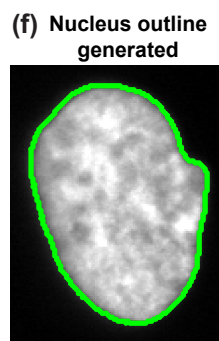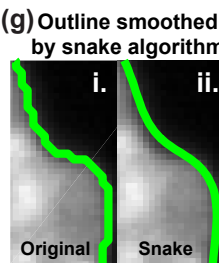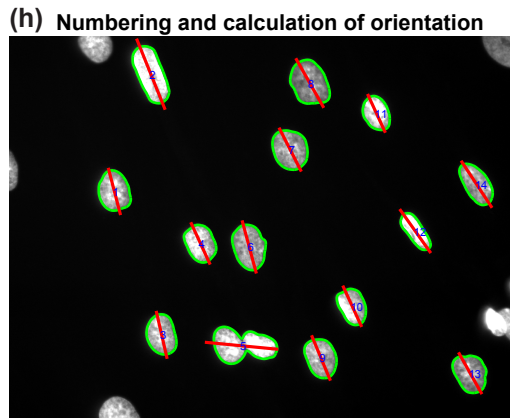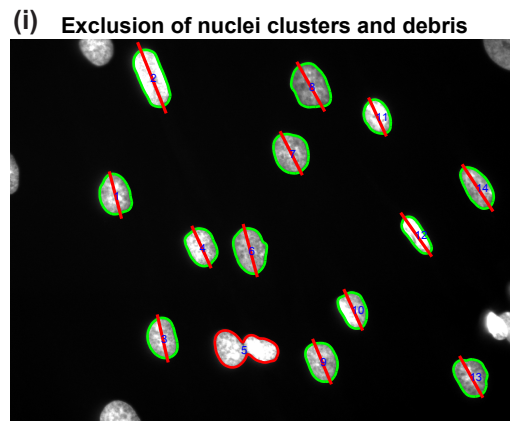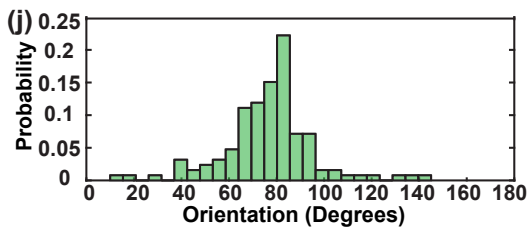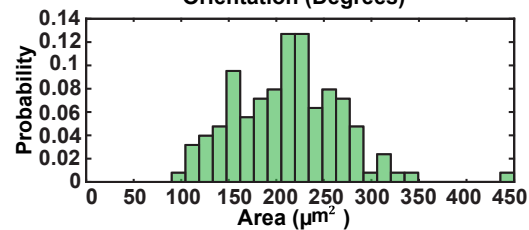

Supplement: S4 Fig — a Original grayscale image; b Binary image generated from first intensity threshold; c Regions of original grayscale image isolated based on detected bodies in binarized image; d Region of original image corresponding to a single nucleus; e Binary image generated from second intensity threshold; f Outline of nucleus generated; g Outline smoothed and given sub-pixel resolution using snake algorithm; h Nuclei labeled and outlined with snake algorithm boundaries; i Overlapping nuclei (red outline) excluded from analysis; j Example data of nuclear area and orientation. Scale bar: 25 μm. (PDF) [file pone.0188256.s012.pdf]

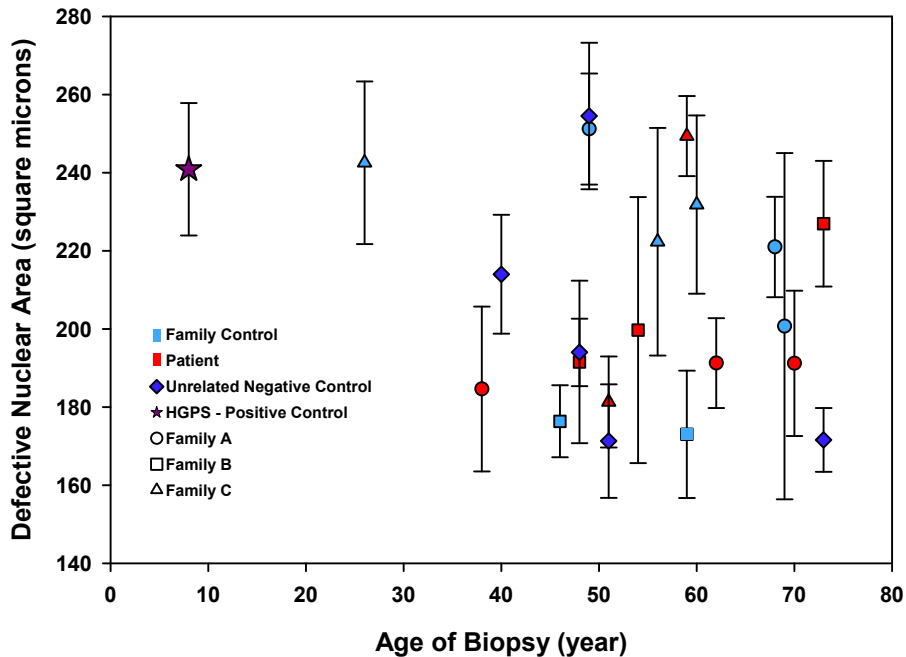

Supplement: S5 Fig — Plot shows no correlations for the data. (PDF) [file pone.0188256.s013.pdf]
